# Exhumation and tectonic history of inaccessible subglacial interior East Antarctica from thermochronology on glacial erratics

**DOI:** 10.1038/s41467-022-33791-y

**Published:** 2022-10-20

**Authors:** Paul G. Fitzgerald, John W. Goodge

**Affiliations:** 1 Department of Earth & Environmental Sciences, Syracuse University, Syracuse, NY 13244 USA; 2 Department of Earth & Environmental Sciences, University of Minnesota Duluth, Duluth, MN 55812 USA; 3 Planetary Science Institute, Tucson, AZ 85719 USA

**Keywords:** Geology, Tectonics

## Abstract

The geology, tectonic history and landscape evolution of ice-covered East Antarctica are the least known of any continent. Lithic boulders eroded from the continental interior and deposited in glacial moraines flanking the Transantarctic Mountains provide rare constraints on the geological history of central interior East Antarctica. Crystallization ages and ice velocities indicate these glacial erratics are not sourced locally from the Transantarctic Mountains but rather originate from the continental interior, possibly as far inland as the enigmatic Gamburtsev Subglacial Mountains. We apply low-temperature thermochronology to these boulders, including multi-kinetic inverse thermal modeling, to constrain a multi-stage episodic exhumation history. Cambro-Ordovician and Jurassic rapid-cooling episodes correlate with significant exhumation events accompanying Pan-African convergence and Gondwanan supercontinent rifting, respectively. Here we show that while Cretaceous rapid cooling overlaps temporally with Transantarctic Mountains formation, a lack of discrete younger rapid-cooling pulses precludes significant Cenozoic tectonic or glacial exhumation of central interior East Antarctica.

## Abstract (web summary)

Glacially transported igneous rocks eroded from the deep interior of ice-covered East Antarctica constrain a remarkable episodic cooling and exhumation history that helps unravel its mysterious tectonic evolution over the last 500 million years.

## Introduction

The geology and subglacial topography of East Antarctica presently covered by the thick East Antarctic Ice Sheet remain enigmatic but vitally important with respect to constraining the tectonic and landscape evolution of the continent. This includes understanding supercontinent assembly, the origin of the high-standing intra-cratonic Gamburtsev Subglacial Mountains, and the inception and stability of the ice cap in response to climate transitions^1,2^. Despite its tectonic and climatic importance, evidence about the subglacial geology of the >99% of East Antarctica covered by ice^3,4^ is largely dependent on indirect methods. Ground, airborne and satellite-based geophysical data (mainly radar, seismology, and potential field data) provide important first-order constraints on sub-ice topography^3^, lithospheric structure^5–11^, sub-ice geology^12^, cryptic tectonic boundaries^13^, and supercontinent relationships^1,14^. Geophysical data interpretation of sub-ice geology often relies on assumed rock properties but is aided by comparison with the geology of ice-free regions and adjacent continents (pre-supercontinent breakup reconstructions), and in the future may be addressed with complementary emerging technologies for directly sampling subglacial bedrock using rapid drilling^15^. To determine the composition, age and thermal history of ice-covered parts of East Antarctica, analyses of detrital materials sourced from under the East Antarctic Ice Sheet provide important constraints on sedimentary provenance and, via geo- and thermochronologic techniques, help constrain the age of interior geologic terranes as well as their cooling/exhumation histories^16–21^.

**Fig. 1:**
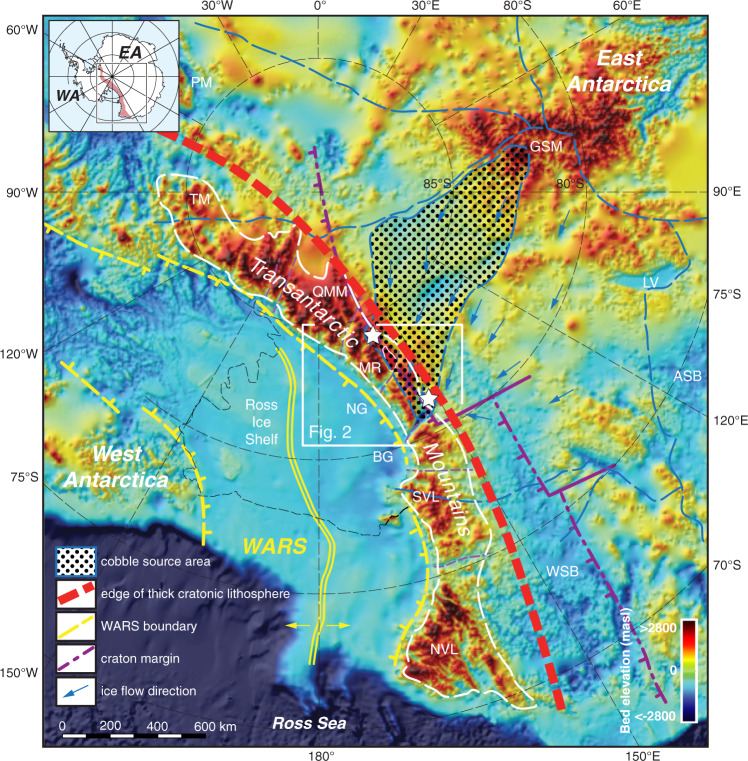
Map of subglacial topography of central East Antarctica showing context of study samples. Sample sites on the inland flank of the TAM shown by white stars. Topography is in meters above sea level, after Bedmap2^3^. Shown are interior subglacial mountains and basins, drainage divides (dashed blue lines) and ice-flow directions within the overlying East Antarctic Ice Sheet. Blue line outlining heavy dots shows source area of glacial moraine cobbles inferred from ice-flow velocity field^22^. White box shows location of area in Fig. 2. Abbreviations: ASB Aurora Subglacial Basin, BG Byrd Glacier, GSM Gamburtsev Subglacial Mountains, LT Lambert Trough, LV Lake Vostok, MR Miller Range, NG Nimrod Glacier, NVL northern Victoria Land, PM Pensacola Mountains, QMM Queen Maud Mountains, SVL southern Victoria Land, TM Thiel Mountains, WSB Wilkes Subglacial Basin. Purple line shows inferred edge of Neoproterozoic rift margin defining the cratonic edge of East Antarctica^14^. Bounding structures of the West Antarctic rift system (WARS) shown in yellow^68^. Heavy dark-orange dashed line shows the approximate edge of thicker cratonic lithosphere of East Antarctica relative to the TAM and the West Antarctic rift system that marks a contrast in exhumation patterns.

**Fig. 2:**
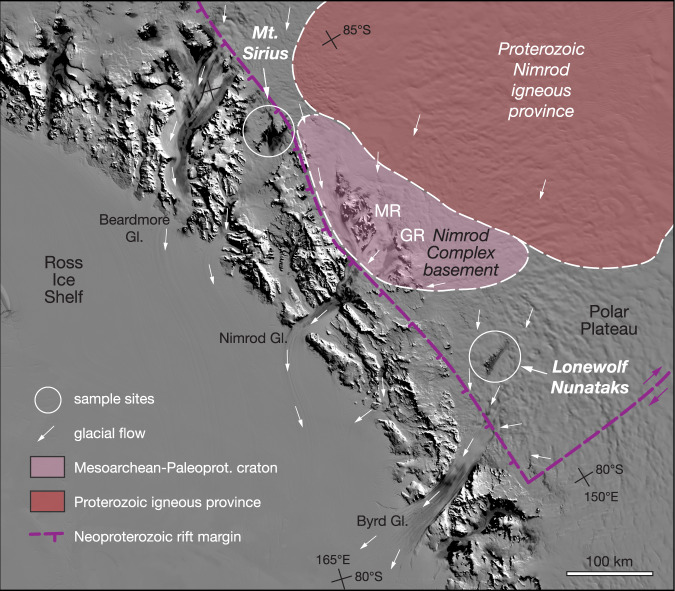
Shaded-relief satellite image with sample sites at Mt. Sirius and Lonewolf Nunataks. Sampled glacial erratics originate from inboard (upstream) of the TAM^22,69^. Mesoarchean and Paleoproterozoic basement (pink) defined by rock outcrop of the Nimrod Complex in the Miller (MR) and Geologists (GR) ranges^70^, together with subglacial geologic terrain defined by aeromagnetic anomalies^14^. Proterozoic Nimrod igneous province (brown) is defined by high-amplitude, positive subglacial magnetic anomalies that resemble those of Proterozoic crustal provinces in Laurentia and Australia^14^; there are no rocks of this province exposed, but it may be part of the source area for the igneous erratics studied here^22^. Purple hachured line marks edge of the Neoproterozoic rifted cratonic margin of East Antarctica. White arrows show modern ice-flow directions^65^. Base image is from the MODIS radiometer Mosaic of Antarctica^22^.

In this work we apply low-temperature thermochronology—apatite fission track (AFT) thermochronology and apatite (U-Th)/He (AHe) dating—to selected boulder to cobble-sized whole rock glacial erratics collected from high-elevation moraines adjacent to Lonewolf Nunataks and Mt. Sirius on the inland flank of the Transantarctic Mountains (TAM). Sample sites are near the heads of Byrd and Beardmore glaciers that drain ice from central East Antarctica through the TAM^22^ (Figs. 1 and 2). These glacial erratics originate from previously unrecognized Proterozoic crust hidden beneath the East Antarctic Ice Sheet (see Methods below) in the interior of the continent, as shown by two independent lines of evidence established in a previous study^22^ using zircon U-Pb crystallization ages and subglacial magnetic anomalies. Results therefore directly constrain the cooling and exhumation history of parts of interior East Antarctica upstream of the sample locations, including perhaps the southwest flank of the Gamburtsev Subglacial Mountains. Samples are large enough (several kg by weight) that processing for mineral separation is the same as for outcrop samples. As all minerals originate from the same erratic boulder this means that multi-mineral thermochronology can yield a more complete thermal history for each sample. That we are analyzing boulders or cobbles is especially important for AFT thermochronology as this utilizes the strength of the method, taking advantage of kinetic parameters such as confined track length distributions and composition proxies that must be collected from many hundreds of grains within each sample^23^. These data along with complementary AHe dating provide inputs to multi-kinetic inverse thermal modeling that constrain temperature-time (*T–t*) histories. Significantly, it avoids a simple closure temperature interpretation for the thermochronology data. In this study our results provide unique constraints on the Phanerozoic thermal history and tectonic evolution of cratonic East Antarctica, a history that overlaps that of the adjacent TAM but with significant differences.

## Results

### Apatite fission track data and (U-Th)/He ages

We determined AFT and AHe ages for seven granitoid samples having zircon U-Pb crystallization ages ranging from ~1.2 Ga to >2.0 Ga (Table 1). AFT central ages range from 479 ± 21 (± 1σ) to 80 ± 6 Ma and all pass the χ^2^ test with low dispersion, indicating single age populations. Mean track lengths range from 12.1 to 13.7 µm, with standard deviations that range from 1.3 to 2.3 µm (Supplementary Data S1 and S2a), indicative of complex thermal histories involving partial annealing and/or episodic cooling^24^. Mean AHe ages range from 382 to 68 Ma with considerable age dispersion in some samples that can be correlated with cooling rate (Supplementary Data S3). As discussed below, AHe single grain ages from cratonic regions are typically over-dispersed and may even be inverted with respect to AFT ages as small variations in the diffusivity of He in apatite—due to, for example, variable grain size, [eU] (effective uranium), and zonation—are magnified by typical cratonic slow protracted thermal histories^25–27^. Within these otherwise unrelated glacial erratics, the preservation of a consistent low-temperature thermal history indicative of multi-stage episodic cooling and exhumation spanning the early Paleozoic to Cretaceous is remarkable and significant.

**Table 1:** Summary of geochronology and thermochronology ages

Sample	Zircon U-Pb age (Ma) (±1σ)	AFT age (Ma) (±1σ)	Mean Length Std. Dev. (µm)	Mean AHe age^d^ (Ma) (±1σ)
**Group 1**^a^				
10LWB-4.5^c^	1848 ± 13	406 ± 20	12.6 (1.7)	220 ± 146
10LWB-4.1^c^	1865 ± 9	479 ± 21	13.4 (1.2)	382 ± 23
10LWA-8.1^c^	2015 ± 12	376 ± 13	12.1 (1.9)	152 ± 5
**Group 2**^b^				
10LWA-11.1^c^	1213 ± 14	131 ± 4	13.3 (2.0)	86 ± 9
10MSA-2.3^c^	1410 ± 10	151 ± 6	13.6 (2.0)	–
10LWB-4.3^c^	1448 ± 5	80 ± 6	13.0 (2.3)	68 ± 26
10MSA-3.5^c^	1508 ± 12	101 ± 5	13.7 (1.8)	101 ± 17

^a^Crystallization ages 1.8–2 Ga, upper crustal rapid cooling events in the Cambro-Ordovician (ca. 500 Ma) and mid-Cretaceous (ca. 125–90  Ma).

^b^Crystallization ages 1.2–1.5 Ga, upper crustal rapid cooling events in the Jurassic and mid-Cretaceous (ca. 100 Ma).

^c^Locations: *LW* Lonewolf Nunataks, *MS* Mt Sirius.

^d^Apatite (U-Th)/He mean ages and uncertainties are calculated using the method of Ault et al.^62^.

### Inverse thermal models and *T–t* evolution of interior East Antarctica

**Fig. 3:**
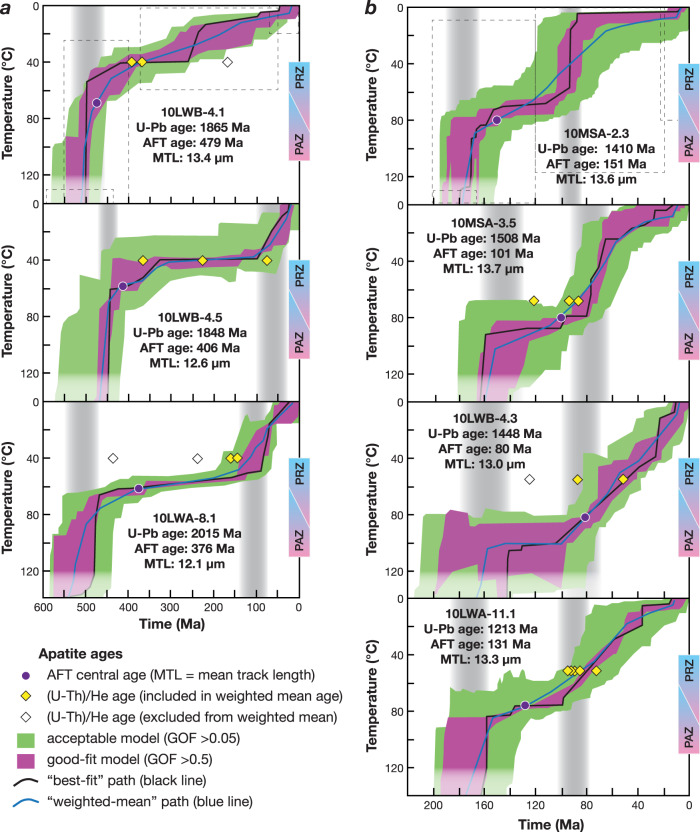
Inverse thermal histories for igneous boulders and cobbles from interior East Antarctica. Models are divided by similar thermal histories into groups 1 (**a**) and 2 (**b**). Light-gray dashed boxes indicate *T*–*t* model constraint boxes (applied to all models in each group). Shown are good-fit (magenta) and acceptable-fit (green) *T*–*t* envelopes based on goodness-of-fit criteria (GOF > 0.5 and 0.05, respectively) that are determined by the probability of failing the null hypothesis that model and measured data are different. In general, a value of ≥0.05 (default value) is considered not to fail the null hypothesis, reflecting an acceptable fit between model and data^28^. Semi-transparent regions are unconstrained (i.e., >120 °C and older than the oldest modeled track). PAZ—partial annealing zone; PRZ—partial retention zone. AFT central ages shown by purple circles, and single-grain apatite (U-Th)/He ages shown by diamonds (yellow where they lie within the good or acceptable envelopes, white where they do not). AHe single grain ages are plotted with respect to their closure temperature determined using CLOSURE, which uses grain size and cooling rate^66^. We identify outlier single grain ages as those that lie outside the good-fit or acceptable-fit *T*–*t* envelopes, likely due to many well-documented factors (e.g., poor grain quality, variable grain size, variable inter- or intragrain effective uranium [eU] zonation, or the presence of uranium-rich inclusions) with single-grain age dispersion magnified by slow cooling and long-term residence in an AHe partial retention zone^26,27,61^.

Multi-kinetic inverse thermal modeling^28^ using AFT data as the primary input, supported by AHe ages (see Methods and Supplementary Data S5) from each individual boulder, provide constraints on the *T-t* histories of crust in the continental interior. Note that the modeling parameters allowed cooling and/or reheating paths throughout geologic time to be explored (see Methods and Fig. 3). The resulting thermal models define two distinct sample groups. Group 1 samples have Paleoproterozoic zircon U-Pb crystallization ages of ~2.0–1.8 Ga and AFT ages ranging from 479 to 376 Ma; their thermal models indicate an initial period of rapid cooling in the early Paleozoic. In contrast, group 2 samples have Mesoproterozoic zircon U-Pb crystallization ages of ~1.5–1.2 Ga and AFT ages of 151–80 Ma; their thermal models indicate Jurassic and younger episodes of rapid cooling. Aside from contrasting thermal histories and U-Pb zircon ages, there are no distinctive differences in lithologies between group 1 and 2 samples in that both comprise a mix of undeformed to variably foliated granitoids. Nor are there any systematic differences in AFT or AHe data between the groups except that group 1 samples are older and hence have a higher spontaneous track density, as would be expected. The different thermal models combined with their different U-Pb ages indicate the boulders likely originated from two distinct geologic source terrains.

Our new inverse thermal modeling points to rapid cooling episodes within the interior of the East Antarctic craton starting at ~500, ~180, and ~125–90 Ma (Fig. 3). The older Paleoproterozoic group 1 samples experienced rapid early Paleozoic cooling (ca. 500 Ma), with some samples also cooling rapidly in the mid-Cretaceous. These samples experienced long periods of slower cooling or long-term residence in an AHe partial retention zone (up to ~350 My) and as a result there is significant dispersion of AHe single grain ages (e.g., samples 10LWB 4.5 and 10LWA 8.1). None of the group 1 thermal models shows evidence of partial resetting due to burial by Beacon Supergroup (Devonian–Triassic) sediments or younger strata, or partial resetting due to Jurassic basaltic magmatism (Ferrar Large Igneous Province—FLIP)^29^. That we see no partial thermal overprint due to sedimentary burial or basaltic magmatism is consistent with paleogeographic reconstructions for the Beacon Supergroup using paleocurrent evidence. Those reconstructions indicate that deposition was confined to elongate basins subparallel to the modern TAM with high-standing terrain on either side, and also that Jurassic magmatism was focused along the axis of the present-day TAM^30^ and did not extend far inboard of the TAM. The lack of evidence for partial annealing since the Jurassic, as apparent in the models, has possible implications for the extent of the hypothesized Transantarctic or Victoria Basin^31–33^, as discussed below.

**Fig. 4:**
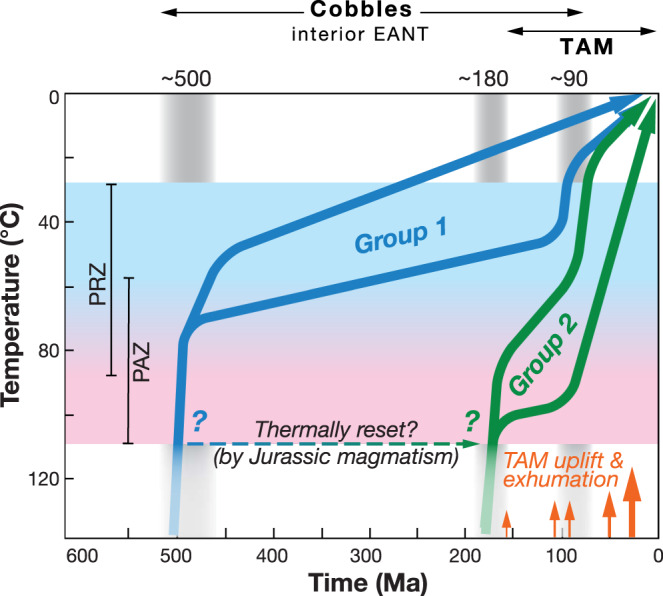
Synoptic temperature-time plot showing Phanerozoic thermal histories of glacial boulders and cobbles from interior East Antarctica. Synoptic inverse thermal histories of groups 1 (blue) and 2 (green) (from Fig. 3) show episodes of rapid cooling highlighted with gray vertical bands. Periods of TAM uplift and exhumation are shown for reference with red arrows, with size of arrow indicating approximate relative amount of exhumation, for example, taken from previous studies^26,32,35–48^.

The younger Mesoproterozoic group 2 samples experienced a more recent Mesozoic history with initial rapid cooling during Jurassic times, although this is not well defined, possibly beginning earlier in some models (ca. 200 Ma) and later in other models (ca. 150 Ma). Long-term residence in an AFT partial annealing zone (PAZ) followed, then rapid cooling since mid-Cretaceous time (ca. 100 Ma with some variability in timing) and sharing that Cretaceous cooling history with one group 1 sample. In the group 2 models it is apparent that those samples with slightly faster cooling paths (e.g., sample 10LWA-11.1) show considerably less AHe single-grain age dispersion (as discussed above and as would be expected). It is also possible that group 2 samples underwent Paleozoic rapid cooling and were then subsequently completely reset by Jurassic thermal events (Fig. 4). However, there is no evidence in the models for partial thermal resetting in the Jurassic and there is some variability in the timing of that initial Jurassic rapid cooling as compared to the well-defined temporal constraints for Jurassic basaltic magmatism at 183 Ma^34^. This further indicates groups 1 and 2 boulders were eroded from two distinct geologic terrains.

### Comparison with low-temperature thermochronology from the TAM

There is a large body of low-temperature thermochronology data from the TAM that we can compare to the new data from interior East Antarctica^26,32,35–48^. The TAM are rift-flank mountains adjacent to the West Antarctic rift system, reaching elevations exceeding 4000 m, but overall the geology of these mountains is relatively straightforward^30,49^. Basement is comprised of Proterozoic–Cambrian metamorphic rocks and Cambrian–Ordovician igneous rocks of the Granite Harbour Intrusive Suite. The basement was deformed during the Ross Orogeny that preceded and accompanied intrusion of these granitoids. Following the Ross Orogeny the TAM underwent significant exhumation to produce the low-relief Kukri Erosion Surface, which is unconformably overlain by Devonian–Triassic sediments of the Beacon Supergroup. The initial breakup of the supercontinent Gondwana in the Jurassic is marked by the FLIP – intrusion of thick sills of Ferrar Dolerite and its extrusive equivalent, the Kirkpatrick Basalt, at 182.8 ± 0.03 Ma^34^. A ca. 160 Myr gap in the on-land TAM geologic record follows until late Cenozoic alkaline volcanism of the McMurdo Volcanic Group^50^ and glacial deposits associated with the Oligocene-recent development of the East Antarctic Ice Sheet^2,51^. Presently, near-flat lying strata of the Beacon Supergroup dip gently under the East Antarctic Ice Sheet with the front of the range marked by normal faults usually striking subparallel to the range front, and in places escarpment retreat is documented^43,48^. Generally, the youngest thermochronology ages representing the greatest (and more recent) exhumation lie along the rift flank adjacent to the coast, with ages getting older with increasing elevation and reflective of less exhumation along the inland flank of the TAM^42^. Overall, compared to young active orogens, exhumation is very slow, but episodes of faster exhumation have occurred. Notably there was a significant increase in exhumation rates in the Late Eocene (~35 Ma) related to rift flank development and formation of the modern-day TAM, also penecontemporaneous with the onset of glaciation in Antarctica^2,35,40,51^. In southern Victoria Land, Early Eocene exhumation preceded Late Eocene exhumation^26,40,43^. There were also exhumation episodes in the Early and mid-Cretaceous^52^, possibly related to plateau collapse of the TAM^53^.

Numerous workers have used the stratigraphy of the TAM, including the elevation of the Kukri Erosion Surface along with the local thermochronology age-stratigraphy (because age varies systemically with elevation), to document the structure of the range, spatial and temporal patterns of exhumation, and paleo-geothermal gradients^37,45,47,48^. Mid-Mesozoic basaltic magmatism (FLIP) signaling the initial breakup of Gondwana was a significant thermal event along the TAM with thermal effects on basement and sedimentary strata. An increase in regional and local geothermal gradients may have reset or partially reset AFT systematics in places where Jurassic magmatism is present. However, this does not apply to rock samples nearer the front of the TAM that were exhumed in the Eocene, as their crustal depth meant they were at temperatures higher than those defining the base of the AFT PAZ. At places on the inland flank of the TAM, samples have not been reset at all; for example in the Miller Range where AFT ages of 250–340 Ma collected over ~600 m of relief represent an exhumed post-Ross PAZ^46^. At one location in the Eisenhower Range of northern Victoria Land, samples collected over elevations of 200–2400 m have AFT ages ranging from ~32–175 Ma, with the uppermost sample lying close to and beneath a large basaltic lava flow that had been erupted onto a subaerial erosion surface^33^. Thermal modeling of these Eisenhower Range data suggested possible *T*–*t* paths of either slow cooling prior to Jurassic magmatism (compatible with formation of that erosion surface) or slow reheating (compatible with the later stages of Beacon Supergroup deposition) followed by reheating—initially rapid, then slowing—associated with development of a large sedimentary basin (the Transantarctic or Mesozoic Victoria Basin)^31–33^. The extent of this basin has been suggested to extend over the entire TAM, the Ross Embayment, most of Zealandia, some of southeastern Australia, and parts of interior East Antarctica including the catchment region for our samples. In those Eisenhower Range models, rocks were subsequently cooled (exhumed) during Late Eocene-Oligocene rift-flank formation. None of our boulders eroded from interior East Antarctica show evidence of partial resetting that would be compatible with the location and formation of this postulated Mesozoic Victoria Basin, although basin strata may not have been thick enough to cause partial resetting. However, the Jurassic and Cretaceous cooling (exhumation) evident in our models could conceivably have supplied detritus to any regions of deposition downstream from the catchment area, including this postulated basin. We note that although we have a small number of samples and Antarctica is a large continent, any evidence for exhumation (in our catchment region or along the TAM) is incompatible with suggested burial due to sediment deposition in the same location on the scale proposed.

## Discussion

The preservation of an ancient Paleozoic and Mesozoic, but not including a Cenozoic, low-temperature cooling history from central East Antarctica is significant. The three recognized periods of cooling in the early Paleozoic, the mid-Jurassic and Late Cretaceous correspond to well-documented Phanerozoic tectonic and magmatic events in Gondwana continents. As such, our new cooling history data show both preservation of distinctive multi-stage cooling within samples, replication of cooling episodes between samples, and that cratonic East Antarctica responded dynamically to episodic regional geologic and tectonic events. Rapid cooling in Cambro-Ordovician times shown by our group 1 samples may be associated with regional Pan-African events representing Gondwana amalgamation that is evident around much of East Antarctica’s margin^12^, and/or with Andean-style Ross Orogen convergent-margin tectonism along the edge of continental East Antarctica and underlying the modern TAM^49^. It is not possible to differentiate these two roughly coeval orogenic signatures based solely on the ~500 Ma rapid cooling defined by our good-fit thermal model envelopes. However, attributing the cooling patterns to intracratonic Pan-African events is consistent with the exotic origin of the cobbles, reconstructions that place Pan-African belts across the catchment area^12,54^, and the limited extent of Ross Orogen activity beneath the East Antarctic Ice Sheet^14,55^.

A Jurassic cooling signature in our group 2 samples corresponds temporally to well-known FLIP magmatism that occurred over a very brief period of geologic time at 183 Ma^34^, and can also be correlated to longer-lived lithospheric extension accompanying breakup of the Gondwana supercontinent^30^. However, the rapid cooling occurring between about ca. 180 and 150 Ma is neither well constrained nor uniquely (temporally or spatially) correlated to FLIP magmatism. This cooling is better explained as exhumation accompanying Jurassic rifting and plate margin re-organization, including block rotation/translation and continental extension related to Gondwana breakup, rather than simple post-FLIP thermal relaxation of elevated magmatic isotherms alone. Rapid exhumation associated with post-FLIP rift-related tectonism beginning ca. 165 Ma is recognized in thermochronology data from the Thiel Mountains^44^ and that interpretation is very likely analogous to the modeled Jurassic cooling in these boulders.

Cretaceous rapid cooling initiated at ca. 125 to 90 Ma (Fig. 3) is evident in most models for both groups. The models possibly reflect discrete episodes of both Early Cretaceous exhumation (ca. 125 Ma) and mid-Cretaceous exhumation (ca. 100 Ma) episodes, similar to what is observed along the inboard, less exhumed parts of the TAM^42,43,52^ (Fig. 4). Typically, in inverse thermal models the spread or overlap of good-fit *T*–*t* paths from basement samples that are geographically close together help constrain the precision of the thermal histories. In this case, our boulders may originate from anywhere within the catchment and likely were sourced from distinct geologic terranes, such that the slightly variable Cretaceous signals may be real. In the TAM, Cretaceous exhumation has been linked to collapse of a high-elevation plateau prior to continental extension in the West Antarctic rift system (WARS; Fig. 1), during which time the WARS underwent continental extension and topographic reversal^43,53,56^. The Cretaceous exhumation evident in the East Antarctic erratics may likewise record these events, notably inland plateau collapse, but may also be related to rifting and strike-slip faulting along the East Antarctic rift system that bounds the high-standing Gamburtsev Subglacial Mountains and extends to the Lambert rift^1^.

Definitive evidence for initiation of Cenozoic rapid cooling events is notably lacking in the thermal models, thus significant Cenozoic exhumation appears unlikely in this part of interior East Antarctica. This distinguishes the thermal history of these interior East Antarctic boulders from TAM basement that typically shows evidence of strong Eocene exhumation linked to rift flank formation and escarpment retreat associated with extension in the WARS^43^ and with penecontemporaneous Oligocene onset of glacial erosion during early stages of East Antarctic ice sheet formation^2,35,40,51^. Since Oligocene time, the Antarctic landscape was modified by glacial erosion, enlarging fluvial drainage networks such as in the Lambert trough and paleo-river drainages preserved in the alpine topography of the Gamburtsev Subglacial Mountains^2,19,51,57^, yet glacial scour in our East Antarctic catchment area is thought to have eroded ≤200 m vertically^2^. Ice sheets in East Antarctica were dynamic from Oligocene to mid-Miocene time (ca. 34–14 Ma), likely fluctuating between near-modern to deglaciated states and reaching their current extent as cold-based glaciers by ca. 13.6 Ma^2^, indicating that our cobbles were likely transported to their present location since the Late Miocene^58^. Despite intriguing best-fit pathways suggestive of rapid cooling at ca. 30–40 Ma in two group 2 models (samples 10LWB-4.3 and 10LWA-11.1; Fig. 3b), the good-fit envelopes do not show rapid cooling at that time. Thus, there is no definitive evidence of rapid cooling beginning ca. 35 Ma that we could attribute to exhumation due to rift-flank tectonics and/or the onset of glacial erosion. We surmise that evidence from our samples for only Cretaceous and older exhumation in interior East Antarctica indicates a contrast between thick cratonic lithosphere of East Antarctica, largely stable since the Cretaceous, and those regions affected by Cenozoic extension in the uplifted TAM and adjacent WARS (Fig. 1).

**Fig. 5:**
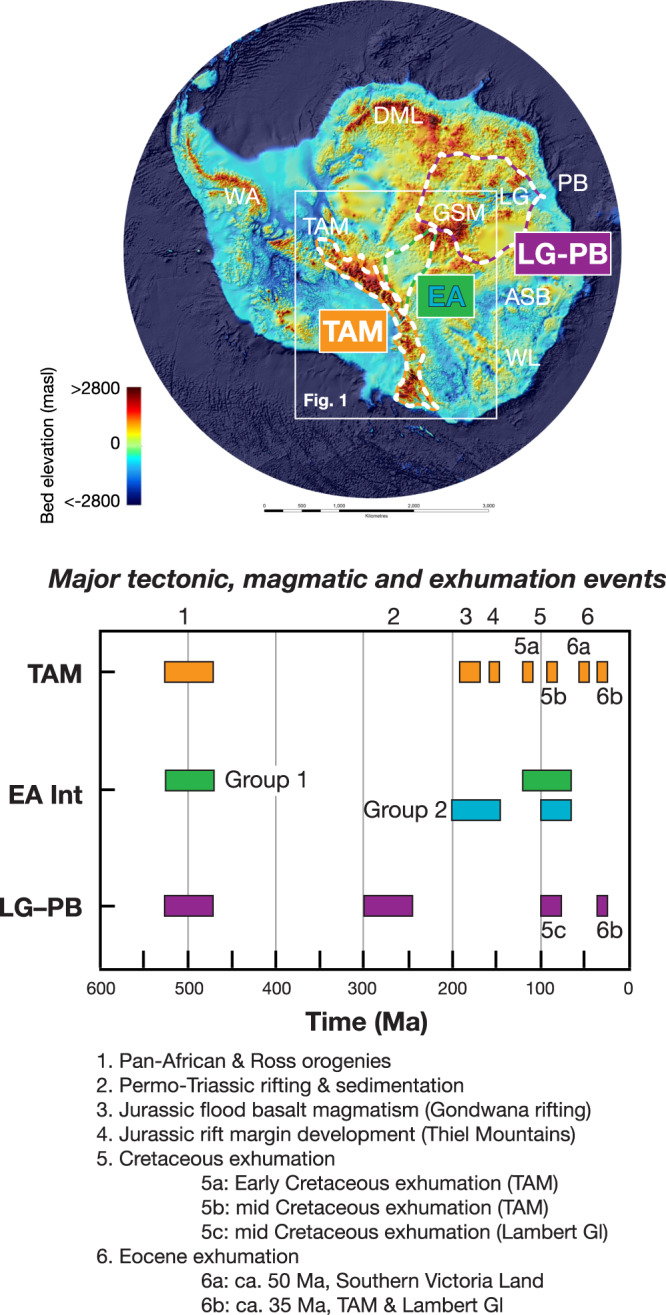
Summary of exhumation events from interior East Antarctica, the TAM and the Lambert Glacier-Prydz Bay region. See text for discussion. Subglacial topography is after Bedmap2^3^; white box shows area of Fig. 1. Dashed lines outline the TAM and show catchment regions for interior East Antarctica (green and white line—EA) and Lambert Glacier-Prydz Bay region (purple and white line—LG-PG), both of which use detrital geo- and thermochronology to constrain the exhumation history of terrain under the East Antarctic Ice Sheet. Abbreviations: ASB Aurora Subglacial Basin, DML Dronning Maud Land, GSM Gamburtsev Subglacial Mountains, WL Wilkes Land, LG Lambert Glacier, PB Prydz Bay, WA West Antarctica.

As synthesized above, our new data and interpretations compare well with the existing geologic and tectonic record of the known Phanerozoic history of East Antarctica (Fig. 5). The new data also compare well with previous detrital studies seeking to constrain the exhumation record of interior East Antarctica. The data from boulders and cobbles however, provide more definitive results than those of detrital studies on sedimentary materials due to the inherent complexity of interpreting populations of single mineral grains derived from multiple source terrains with variable thermal and exhumation histories^59^. Firstly, the provenance of sedimentary detritus may be uncertain. With respect to the interior of East Antarctica, it may be unknown or poorly constrained whether the detritus analyzed actually originated from interior East Antarctica, was reworked, or came from nearby exposed outcrops^19,20^. For example, single-grain multi-mineral and multi-method thermochronologic data from moraines in the TAM yielded results that suggest both recycling from the Devonian–Triassic Beacon Supergroup sedimentary strata and erosion of East Antarctic sub-glacial rocks^20^. These data record information relating to pre-Beacon depositional cooling/exhumation of the source area as well as later post-depositional cooling and erosion associated with Gondwana breakup^20^.

Secondly, most detrital dating studies undertaken so far to constrain the thermal and exhumation history of interior East Antarctica are performed on single mineral grains. Therefore, by necessity simple closure temperature assumptions are usually applied to interpret individual ages. For higher-temperature techniques this is useful to constrain crystallization or timing of metamorphism, but for low-temperature techniques may yield ‘time-averaged interior exhumation rates’ that are extremely low and implying, perhaps erroneously, the presence of a landscape that is ancient and has not changed for 100 s of million years^60^. In ODP cores offshore of Prydz Bay, for example, multi-method dating from single grains of detrital apatite and zircon in Oligocene-Quaternary sedimentary strata yield a relatively complete thermal history for Antarctica inland from Prydz Bay^21^. U-Pb and ^40^Ar/^39^Ar ages on these grains record crystallization ages of ca. 500 Ma, reflecting Pan-African magmatism and accompanying metamorphism, while fission track and (U-Th)/He ages record Permo-Triassic cooling/exhumation (correlated with intra-continental rifting). Decreasing fission track and (U-Th)/He age lag-times in Oligocene-Miocene strata suggest increasing erosion rates (initial glacial erosion), followed by decreasing lag-times after the Late Miocene, interpreted as due to cold-based and less erosive glaciation^21^. In a complementary study, Thomson et al.^19^ used a similar approach, undertaking detrital thermochronology on Oligocene strata deposited offshore during expansion of the East Antarctic Ice Sheet, as well as from onshore moraines. They interpreted single-grain age distributions as indicative of Permo-Triassic cooling/exhumation, with later localized glacial erosion since ca. 34 Ma in the Lambert Glacier region, but they also interpreted a discrete Cretaceous AFT age peak (at ~115 Ma) as originating from Kerguelen plume rocks lying well offshore.

Thirdly, as mentioned above, some low-temperature methods applied to landscape evolution, notably AHe dating, yield high-precision single-grain ages from the same sample but often with overdispersion of ages well in excess of analytical uncertainty^25,26,40,61,62^. Overdispersion is especially common for cratonic rocks where very slow erosion rates (and hence cooling rates) mean that the long-term residence in a partial retention zone accentuates age dispersion^25–27,40,61^. AHe ages on single grains sourced from a cratonic region such as East Antarctica are very likely to also have overdispersion and therefore add uncertainty to the interpretation of any detrital data.

In summary, glacial erratics eroded and transported by the East Antarctic Ice Sheet, then stranded in moraines along the inland flank of the TAM, uniquely preserve Phanerozoic cooling histories representative of interior cratonic East Antarctica. Their cooling histories reflect episodic exhumation separated by long periods of relative tectonic and thermal stability (Fig. 4). Cambrian–Ordovician cooling appears related to late Gondwana supercontinent amalgamation and late-stage post-orogenic exhumation. The Jurassic cooling was likely associated with rifting and Gondwana breakup, whereas Cretaceous cooling may be related to plateau collapse linked to continental extension within East Antarctic lithosphere and the West Antarctic rift system or exhumation along the rift flank of the Gamburtsev Subglacial Mountains. The Cretaceous and older cooling/exhumation events correspond to well-known events in the TAM, but the glacial erratics lack evidence for younger cooling events indicative of Cenozoic exhumation of the continental interior due either to tectonic events or enhanced glacial erosion.

If our samples do indeed originate from the flank of the Gamburtsev Subglacial Mountains, their modeled *T*–*t* histories support an interpretation based on detrital mineral ages in sediments cored in Prydz Bay (Fig. 5) that this enigmatic subglacial massif may have an early Paleozoic (Pan African) tectonic origin^60^. However, our data show no evidence that Gamburtsev mountains formation was due to either rapid Permian exhumation (ca. 250 Ma) along the East Antarctic rift system^1^ or to mid-Carboniferous (ca. 320–300 Ma) inversion of an intracratonic superbasin^63^. Similarly, our results are consistent with a study that sampled basement rocks from the flanks of the Lambert rift^64^ in that both constrain Cretaceous exhumation from inverse thermal models. While the mid-Cretaceous exhumation signal from the Lambert Glacier^64^ is not detrital and would not yield a detrital signal with grains of that age as it is constrained by modeling, the possibility of Cretaceous exhumation in this drainage was rejected by later detrital studies^19^. Even if detrital datasets from either side of the Gamburtsev mountains do not give overlapping results, for example we observe no Permo-Triassic cooling in inverse thermal models, that should not negate the possibility of exhumation associated with formation or reactivation of mountain formation during the Permian and/or the Cretaceous^1^. This is because the catchment area for our limited number samples is large, and if the samples are from a higher structural level on the interior side of a rift-flank system they may not yield a rift-formation exhumation signature in the Permian.

The evidence from low-temperature thermochronology presented here strongly suggests that within our catchment region there are two distinct crustal age provinces with Paleoproterozoic and Mesoproterozoic igneous belts, each with different cooling histories, and sampled by glacial flow. Such a conclusion is not possible based solely on zircon U-Pb crystallization ages. Group 2 samples may have a stronger affinity with the TAM based on the timing of Jurassic and Cretaceous cooling/exhumation, yet a difference in Cenozoic thermal history compared to the TAM indicates a contrast between inboard stable lithosphere of the East Antarctic craton and extended lithosphere adjacent to and within the WARS (Fig. 1). Such an inference is testable by further systematic study of high elevation glacial erratics from different catchments. However, after sampling moraines distributed over >1500 km along the inland flank of the TAM^22^, Precambrian erratics were found at only a few sites between the Byrd and Beardmore glaciers, including the two sites at Lonewolf Nunataks and Mt. Sirius that provided samples for this study. The presence or absence of exotic cratonic material appears to be controlled by variations in glacial transport and subglacial geology, both of which are not yet well understood in East Antarctica^12,22^. The paucity of glacially derived cratonic debris is not unexpected, however, given that the only exposed Precambrian basement rocks along the entire TAM margin of East Antarctica between Terre Adélie and the Shackleton Range are found in the Miller Range and Geologists Range of the central TAM. Further tests of our interpretations regarding a low-temperature cooling and exhumation history of cratonic East Antarctica may therefore be hampered by a lack of suitable glacial samples. In the future, our interpretations will be more directly testable by thermochronology on rock cores recovered by deep drilling into ice-covered bedrock.

## Methods

### Sample sites and characteristics

The igneous cobble and boulders analyzed are part of an earlier study^22^ where surficial moraines on the high-standing inland flank of the TAM were sampled to obtain glacially eroded and transported rock material derived from beneath the East Antarctic Ice Sheet. The timing of moraine deposition is not known but is certainly younger than mid-Miocene and likely ranges in age from Pleistocene to modern^58^. Both igneous and metamorphic lithologies were previously sampled at sites stretching >1500 km from north of the Dry Valleys to south of Reedy Glacier in the southern TAM. Heterolithic glacial erratics from cobble to boulder size with rounded shapes and surficial glacial striations indicative of glacial transport were collected from moraines and active blue-ice streams and then sorted into different lithotypes. Goodge et al.^22^ culled an initial set of >300 samples based on petrography and in situ U-Pb zircon geochronology to a subset of 40 granitoid igneous rock samples that underwent detailed petrographic, geochemical, geochronologic and isotopic study. Ross Orogen-age granitoids (ca. 500 Ma) were excluded from further analysis in order to focus on samples representative of interior East Antarctica, ultimately yielding 22 samples with zircon crystallization ages >1.2 Ga. Of this group, seven samples with good quality apatites and a range of zircon crystallization ages were selected for low-temperature thermochronology for this study (Table 1).

### Provenance of the cobbles and drainage region

The granitoid cobbles analyzed here were selected from a larger suite of glacially transported igneous erratics that yielded zircon U-Pb crystallization ages defining magmatic events at ∼2.01, 1.88–1.85, ∼1.79, ∼1.57, 1.50–1.41, and 1.20–1.06 Ga^22^. None of these granitoid age populations are known in exposed Mesoarchean to Paleoproterozoic basement of the nearby TAM (Nimrod Complex) or even the Terre Adélie Craton lying on the other side of the craton^22^. The discrete age populations within this suite of glacial erratics also have distinctive zircon δ^18^O and initial εHf isotopic compositions^22^. This indicates that crust of East Antarctica contains heterogeneous igneous sources and must represent previously unrecognized Proterozoic igneous provinces hidden beneath the East Antarctic Ice Sheet, subsequently eroded and transported by the ice sheet to these high elevation moraines^22^. Cambro-Ordovician Ross Orogen granitoids and Jurassic FLIP magmatism, both of which crop out extensively along the TAM, have distinctive magnetic anomalies that extend only a short distance inland from the TAM^10,11,14,55^. However, an area of distinctive aeromagnetic anomalies lying farther inboard of the TAM (Fig. 2) is interpreted as a Proterozoic igneous province in cratonic East Antarctica^14^ and may in part be the source for some of the Proterozoic granitoid cobbles analyzed here. This subglacial terrain was termed the Nimrod igneous province^14^.

A broader potential source region for these Proterozoic igneous erratics is constrained by modern ice velocities and subglacial topography that define glacial catchments^3,65^ (Fig. 1). Ice velocities of large ice sheets originate with radial flow off high-standing domes such as Dome Argus lying over the highest topography of the Gamburtsev Subglacial Mountains. Despite uncertainties of transport distance and selective glacial erosion^2,51^, the potential catchment extends from the alpine topography of the southwestern flank of the Gamburtsev Subglacial Mountains across lower-lying regions of glacial scour to just upstream of the Byrd Glacier inlet to the sampled moraines (Figs. 1 and 2).

### Low-temperature thermochronology

Low-temperature thermochronology with AFT and AHe is routinely used to resolve upper crustal exhumation and landscape evolution by constraining cooling and, hence, exhumation histories below ~120 °C and ~90 °C, respectively^66^. AFT applied to whole-rock boulders and cobbles^23^ is especially powerful because confined track length distributions, a kinetic parameter, which require measurement from many grains within a sample allow well-constrained inverse thermal models to define best-fit *T*–*t* envelopes. Thermal models can therefore constrain whether samples cooled quickly following igneous intrusion, cooled later due to exhumation, were partially reset following a thermal event (e.g., Jurassic basaltic magmatism) or following burial by sedimentary strata^59^. Note that interpreting models from cobbles or boulders requires a slightly different approach than that for bedrock samples collected from the same region. While bedrock samples commonly share components of the same history depending on their relative crustal level or structural position, and therefore constrain the precision of models for a group of bedrock samples, we should not expect a suite of glacially disaggregated boulders to have the same or similar *T*–*t* histories as they may have been sourced from anywhere in the catchment region. Therefore, slight differences in models derived for different cobbles may be real and should be considered as potentially significant.

### Inverse thermal modeling

A multi-kinetic annealing model HeFTy v.1.9.3^28^ was used with AFT data as the primary input (single-grain ages, *c*-axis projected confined track lengths, composition-proxy D_par_) and complementary constraints from AHe ages using the radiation damage accumulation and annealing model (RDAAM^67^) (Supplementary Data S5). HeFTy uses a Monte Carlo approach to generate *T*–*t* paths through various constraint boxes, followed by statistical tests that determine a goodness-of-fit between the input data and model predictions^28^. Good-fit and acceptable-fit envelopes are based on a goodness-of-fit criteria of >0.5 and 0.05, respectively, determined by the probability of failing the null hypothesis that model and measured data are different. In general, a value of ≥0.05 (default value) is considered not to fail the null hypothesis, reflecting an acceptable fit between model and data^28^. Models are run until there are ten good-fit *T*–*t* paths, typically along with hundreds of acceptable-fit *T*–*t* paths. *T*–*t*constraint boxes (Fig. 3) allow the models to explore *T*–*t* space to test possible scenarios. *T*–*t* model constraint boxes are placed strategically to (i) start the modeling at ages older than the AFT ages and at temperatures well above the sensitivity of the AFT method, and (ii) to allow the model paths to evaluate various geologic scenarios, for example partial thermal resetting during the Jurassic FLIP magmatism, and/or partial thermal resetting due to burial by sedimentary deposits and/or rapid cooling episodes during the Cenozoic. The modeled *T*–*t* path segments are allowed to halve multiple times with randomized episodic changes to either higher temperatures or cooler temperatures to ensure all possible *T*–*t* paths are tested.

## Supplementary information

Supplementary Information

## Data Availability

All low-temperature thermochronology data (AFT and AHe) collected during this study are included in this article and its supplementary information file. This includes AFT counting data, confined track length data, radial plots and (U-Th)/He data with notes on analyzed grains, single-grain age parameter plots (vs. age and [eU]) and detailed information and tables on modeling parameters.
