# Supplementary material for: Exhumation and tectonic history of inaccessible subglacial interior East Antarctica from thermochronology on glacial erratics
Source: Nat Commun. 2022 Oct 20;13:6217. doi: 10.1038/s41467-022-33791-y (PMC9584882; doi:10.1038/s41467-022-33791-y)
Supplement: Supplementary file 1 — Supplementary Information [file 41467_2022_33791_MOESM1_ESM.pdf]

Exhumation and tectonic history of inaccessible subglacial interior East Antarctica from thermochronology on glacial erratics

Paul G. Fitzgerald[1*] and John W. Goodge[2,3]

[1]Department of Earth & Environmental Sciences, Syracuse University, Syracuse, NY 13244 USA

[2]Department of Earth & Environmental Sciences, University of Minnesota Duluth, Duluth, MN 55812 USA

[3]Planetary Science Institute, Tucson, AZ 85719 USA

Paul Fitzgerald (pgfitzge@syr.edu), John Goodge (jgoodge@d.umn.edu)

Supplemental Data Material

Supplementary Data Table S1: Apatite Fission Track Data Table

Supplementary Data S2: Apatite Fission Data

Supplementary Data S2a. Confined track length distributions and radial plots

Supplementary Data S2b: AFT Counting data and measured confined track lengths

Supplementary Data Table S3: Apatite (U-Th)/He Data including notes on analyzed grains

Supplementary Data S4: Apatite (U-Th)/He Single Grain Age Plots (age vs eU, age vs grain size)

Supplementary Data S5: Inverse Thermal Modeling

Supplementary Data References

Supplementary Data Table S1: Apatite fission track data, East Antarctic cobbles, central Transantarctic Mountains region

Sample Number	U-Pb zircon age (Ma)	No. of grains	Fission Track density (x10^6) Standard	Fossil	Induced	$P(\chi^2)$	Rel. Error (%)	[U] (ppm)	Apatite fission-track age (Ma) (± 1σ)	Track lengths (μm) Mean (μm)	Std. Dev. (μm)	Dpar (μm)
***Mount Sirius** (84°07.976'S, 163°15.121'E, 2160 m)*												
10MSA-2.3	1410	25	1.175 (12697)	1.331 (1076)	1.842 (1489)	46	4.9	19.6	151.1 ± 6.4	13.6 (105)	2.0	1.8
10MSA-3.5	1508	28	1.225 (12697)	0.4175 (576)	0.903 (2145)	91	0	9.2	101.4 ± 5.2	13.7 (101)	1.8	1.6
***Lone Wolf Nunataks** (Site A [LWA]: 81°20.186'S, 152°42.461'E, 1590 m; Site B [LWB]: 81°19.545'S, 153°01.445'E, 1595 m)*												
10LWB-4.1	1865	25	1.26 (12697)	1.5275 (1737)	0.698 (794)	75.3	0	6.9	479.3 ± 21	13.4 (95)	1.2	1.5
10LWB-4.3	1448	36	1.302 (12697)	0.1334 (213)	0.388 (2483)	60.2	0.01	3.7	80.2 ± 6.4	13.0 (82)	2.3	1.4
10LWB-4.5	1848	25	1.334 (12697)	3.975 (1511)	2.32 (882)	30.8	10.2	21.7	405.9 ± 20.2	12.6 (100)	1.7	1.5
10LWA-8.1	2015	25	1.408 (12697)	4.7342 (3450)	3.089 (2251)	5.9	9.5	27.4	376.2 ± 13.1	12.1 (165)	1.9	1.7
10LWA-11.1	1213	25	1.429 (12697)	1.7451 (1934)	3.389 (3756)	19.7	7.3	29.6	130.9 ± 4.4	13.3 (114)	2.0	1.7

Sample descriptions with U-Pb zircon age and Hf-O-isotope data provided by Goodge et al. (2017)[1]. Parentheses enclose number of tracks counted (density) or measured (track lengths). Standard and induced track densities were measured on mica external detectors (geometry factor = 0.5), and fossil track densities were measured on internal mineral surfaces. Samples of basement rock were crushed and the apatite crystals separated using conventional heavy liquid and magnetic techniques. Apatite crystals were mounted in epoxy resin on glass slides, ground and polished to reveal an internal surface, and then etched for 20 s at room temperature in 5N HNO_3 to reveal spontaneous fission tracks. Apatite ages were determined using the external detector method and an automated stage. Samples were irradiated at the Oregon State University Nuclear reactor in the slow soaker position B-3 (Thermal column number 5) which has a Cd for Au ratio of 13.6 at the column face. The mounts were counted at a magnification of 1250x under a dry 100x objective. Ages were calculated using the zeta calibration method (zeta = 361 ± 10 for dosimeter glass CN5) following the procedures of Hurford and Green (1983)[2] and Green (1985)[3]. Analytical errors were calculated using the "conventional method"[4]. The chi-square test performed on single-grain data[5] determines the probability that the counted grains belong to a single age population (within Poissonian variation). If the chi-square value ($P(\chi^2)$) is less than 5%, it is likely that the grains counted represent a mixed-age population with real age differences between single grains. The relative error or age dispersion (spread of the individual grain data) is given by the relative standard deviation of the central age. Where the dispersion is low (<15) the data are consistent with a single population, and the mean/pooled ages and the central age converge. Track lengths were measured using "confined" fossil fission tracks using only those that were horizontal[6]. Tracks were measured under a 100x dry objective using a projection tube and a digitizing tablet attached to a microcomputer. Wherever possible, 100 track lengths per sample were measured, the number being less only when insufficient suitable tracks were present in the available apatite.

Supplementary Data S2a: Apatite Fission Track Length Distributions and Radial plots

Track length distributions and radial plots are shown below. For track length distributions the y-axis = n, the number of horizontal confined tracks measured. Also shown is the mean track length, the standard deviation and number of tracks measured for each sample – for example: sample 10MSA 2.3 has a mean length of 13.6 μm with a standard deviation of 2.0 μm. The number of tracks measured is 105. Track counts are not normalized to 100 and distributions are not corrected for angle to the c-axis. Radial plots [7, 8] are generated using the program Radial Plotter 9.4 [9, 10], logarithmic transformation (http://www.london-geochron.com/p/software.html). Radial plots are bivariate scatterplots where the slope of the line is a reflection of the age of the individual grains (with the horizontal line = central age) and the position of each grain age along the x-axis being indicative of the precision of the age (i.e., further to the right indicates a higher precision single grain age). A frequency distribution of the single grain ages is shown on the right reflecting the spread of single grain ages around the central age. Radial plotter also performs a Chi-squared test of the single grain age data to determine age populations, implementing the mixture modeling algorithm of Galbraith and Green [11]). In this case Radial Plotter suggests that there may be two age populations for sample 10LWA 8.1, although this sample does not actually fail the Chi-square test (Chi-squared value is 5.9% and dispersion is 9.5 – see the AFT summary table S1) so this Radial Plotter suggestion of two age populations is not included. For this sample the observed single grain age dispersion is likely due to slightly differing fission track retentivities as a result of slight compositional differences between grains, accentuated because of the long-term residence (ca. 350 Myr) within the AFT partial annealing zone – which is shown in the inverse thermal model for this sample.

Mt Sirius samples

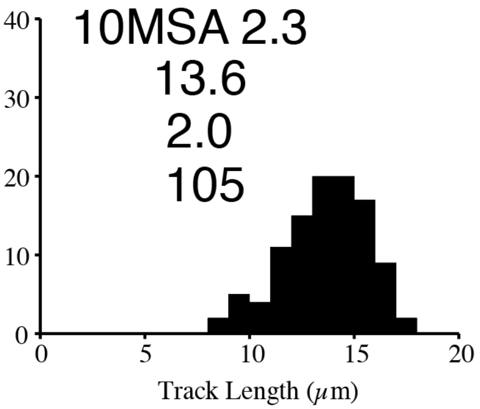

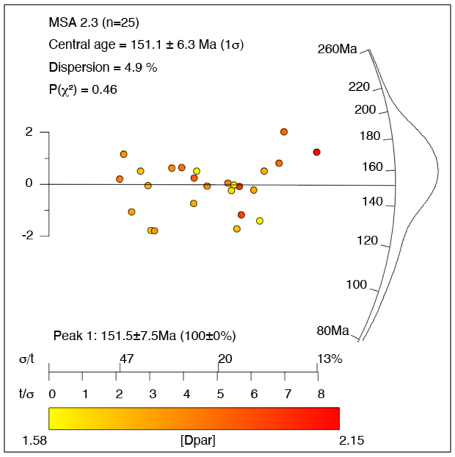

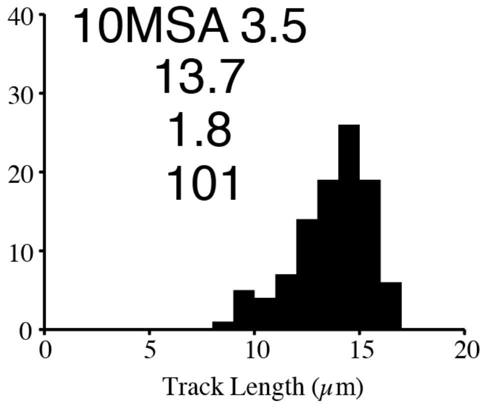

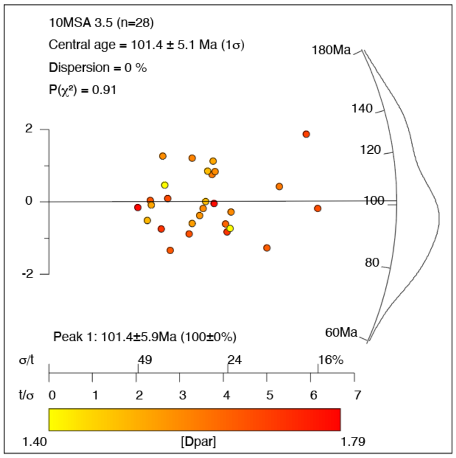

Lonewolf Nunatak samples

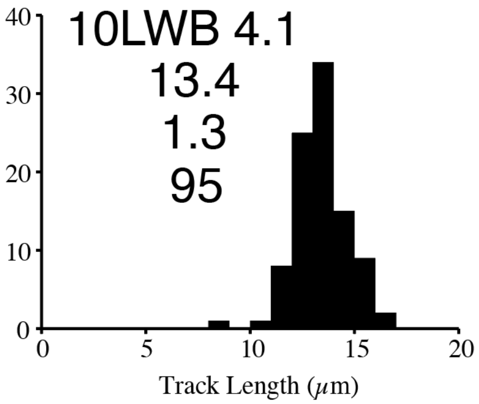

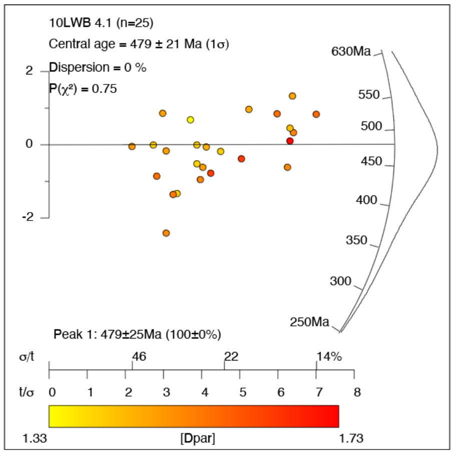

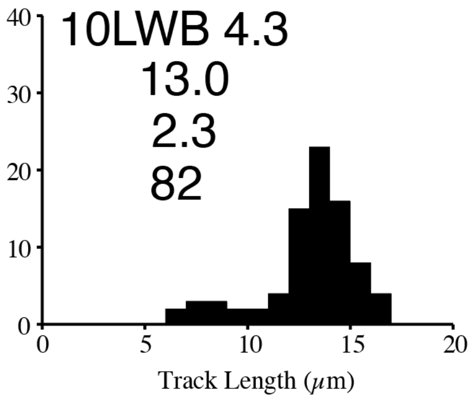

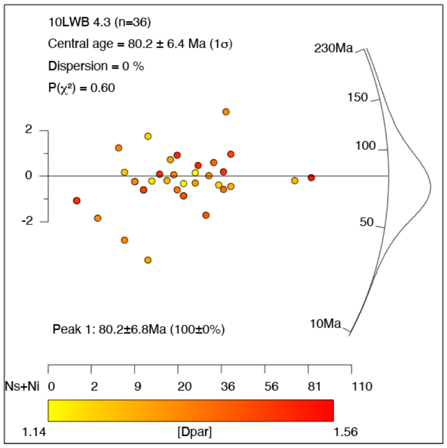

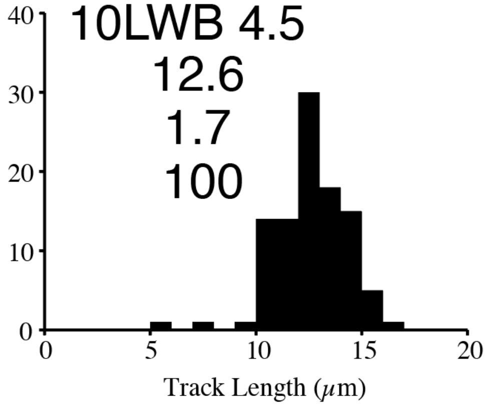
10LWB 4.5
12.6
1.7
100
Track Length (μm)

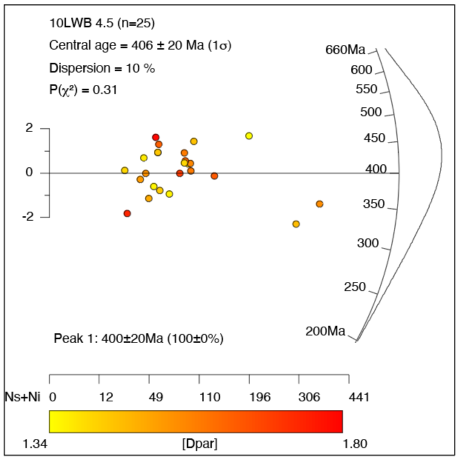
10LWB 4.5 (n=25)
Central age = 406 ± 20 Ma (1σ)
Dispersion = 10 %
P(χ²) = 0.31
Peak 1: 400±20Ma (100±0%)
Ns+Ni
[Dpar]

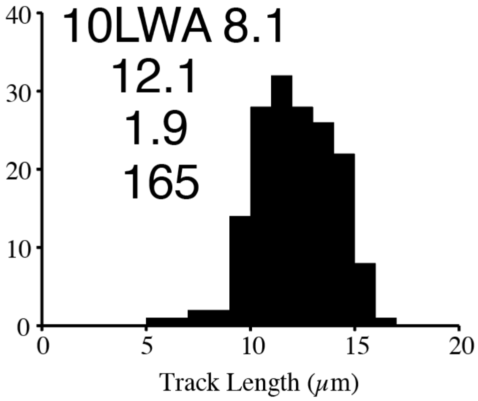
10LWA 8.1
12.1
1.9
165
Track Length (μm)

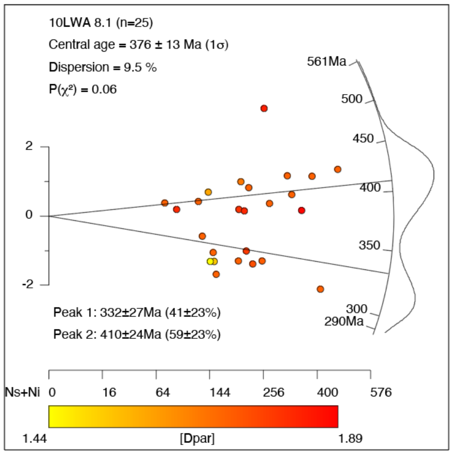
10LWA 8.1 (n=25)
Central age = 376 ± 13 Ma (1σ)
Dispersion = 9.5 %
P(χ²) = 0.06
Peak 1: 332±27Ma (41±23%)
Peak 2: 410±24Ma (59±23%)
Ns+Ni
[Dpar]

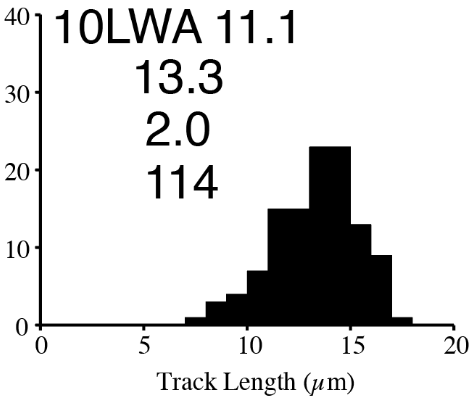
10LWA 11.1
13.3
2.0
114
Track Length (μm)

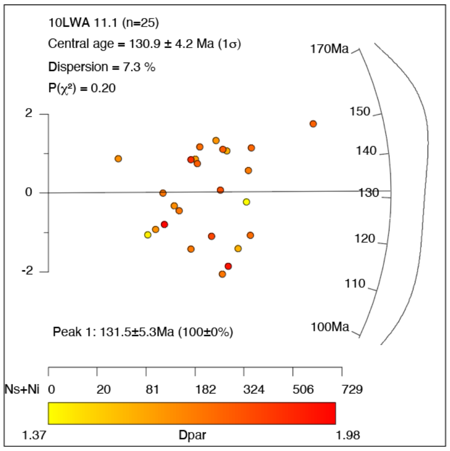
10LWA 11.1 (n=25)
Central age = 130.9 ± 4.2 Ma (1σ)
Dispersion = 7.3 %
P(χ²) = 0.20
Peak 1: 131.5±5.3Ma (100±0%)
Ns+Ni
Dpar

Supplementary Data S2b: AFT Counting Data and Measured Confined Track Lengths

These are listed by sample number; AFT counting data first, then length measurement data – in two columns

10MSA 2.3

zeta	sig zeta	rho-d (N/cm≤)	Nd
**361**	**10**	**1.18E+06**	12697
**Ns**	**Ni**	**Dpar**	
96	99	1.93	
13	32	1.699	
62	107	1.58	
35	43	1.612	
14	34	1.81	
11	9	1.823	
29	34	1.906	
49	67	1.864	
63	90	1.769	
48	90	1.72	
30	49	1.767	
33	43	1.948	
55	77	2.043	
14	16	1.719	
117	138	2.146	
52	72	1.605	
15	21	1.747	
85	104	1.916	
73	93	1.754	
52	88	2.012	
8	10	1.876	
38	53	1.829	
9	19	1.793	
25	29	1.84	
50	72	1.653	

length	angle	Dpar
15.278	42.751	1.662
15.444	45.308	1.65
12.333	75.531	1.59
16.36	42.669	1.59
12.81	79.067	1.59
14.535	50.727	1.59
12.539	72.233	1.59
8.179	48.739	1.499
15.661	67.869	1.709
13.898	14.839	1.86
14.502	68.343	1.998
10.205	29.011	1.593
15.966	17.09	1.732
9.409	25.831	1.666
13.694	59.567	1.841
12.814	64.613	1.935
13.341	41.593	1.751
9.623	60.364	2.276
14.467	88.159	1.862
14.8	67.353	1.526
15.327	72.71	2.055
12.195	9.964	1.767
13.195	48.263	1.648
12.469	62.822	1.94
13.818	89.823	2.012
11.644	49.369	1.666
8.559	75.348	2.079
14.255	70.609	2.188
16.785	41.591	2.188
16.443	37.023	1.868
13.582	79.861	2.011
11.782	57.505	1.642
16.7	65.565	1.642
14.169	52.186	1.642
14.257	62.459	1.642
11.163	31.056	1.922
12.773	37.993	2.235
14.239	63.088	1.794
13.347	37.997	1.636
13.978	7.196	1.535
11.947	86.871	1.719
15.379	87.849	2.125
12.939	83.242	1.75

12.939	60.721	1.493	12.829	59.986	1.854
16.194	53.695	1.674	10.953	29.623	1.777
12.518	42.299	1.941	13.357	48.712	1.775
15.528	17.003	1.699	9.623	64.901	1.797
15.372	45.72	1.378	16.144	37.195	1.673
13.168	84.44	1.811	17.551	36.524	1.817
13.454	79.122	1.494	11.407	59.247	2.017
15.673	87.831	1.933	13.586	62.151	1.955
13.685	61.105	1.82	14.496	67.435	1.579
16.127	42.821	2.079	12.577	46.177	1.474
15.782	82.93	1.694	15.112	69.652	1.567
14.584	19.719	1.973	13.197	75.263	2.095
12.809	82.983	1.852	11.475	59.999	2.138
13.184	79.369	2.245	11.813	65.655	1.977
14.466	16.649	1.762	10.429	59.963	2.044
11.441	27.359	1.92	13.352	76.719	1.679
14.881	78.16	1.818	14.415	70.255	2.205
13.719	61.093	2.028	12.654	66.921	1.63
15.126	78.812	1.827	16.743	39.553	2.056
14.998	32.644	1.932	14.153	79.863	2.044
14.656	37.607	1.859	14.238	71.349	2.229
14.838	78.673	1.928	12.838	44.725	1.548
14.377	67.753	1.769	11.681	82.311	1.68
17.224	18.057	1.869	13.63	59.842	1.927
11.693	60.405	1.871	15.088	4.76	2.071
13.034	44.436	1.966	13.065	65.644	1.628
15.212	63.762	1.388	14.565	76.196	2.127
9.91	32.206	1.783	16.531	27.091	1.21
15.764	21.476	1.733	15.132	67.975	1.836
11.148	65.124	1.669	10.665	26.847	1.577
9.416	26.076	1.71	15.968	60.457	1.494

10MSA 3.5

**zeta**	**sig zeta**	**rho-d (N/cm≤)**	**Nd**	23	37	1.539
				8	18	1.541
**361**	**10**	**1.23E+06**	**12697**	7	19	1.514
Ns	Ni	Dpar		23	61	1.72
8	17	1.673		11	20	1.401
25	58	1.584		12	16	1.58
22	39	1.603		34	95	1.658
11	23	1.684				

6	14	1.79
14	40	1.675
21	46	1.783
17	41	1.569
18	27	1.596
55	123	1.681
57	90	1.699
18	41	1.583
23	58	1.644
21	36	1.505
10	35	1.666
24	62	1.399
42	84	1.613
9	26	1.726
23	40	1.582
15	39	1.552
19	41	1.5

**length**	**angle**	**Dpar**
13.349	66.934	1.949
15.242	79.311	1.655
13.327	45.494	1.65
13.118	66.789	1.475
15.128	85.064	1.63
13.212	61.952	1.825
14.201	86.27	1.825
14.422	56.105	1.825
14.417	59.114	1.825
14.25	58.454	1.381
15.482	57.767	1.464
12.639	89.172	1.464
14.264	62.764	1.467
13.33	73.197	1.467
11.963	67.99	1.49
16.811	4.508	1.461
12.456	22.737	1.461
14.642	81.69	1.397
15.72	44.069	1.584
13.273	61.66	1.584
11.9	78.345	1.418
14.168	30.449	1.356
14.462	54.252	1.603
15.501	37.719	1.603
13.175	62	1.472
13.37	58.774	1.704
14.408	76.89	1.595
15.331	68.809	1.595
14.32	51.498	1.5
13.598	32.026	1.5
14.853	70.436	1.449
12.499	72.996	1.449
16.53	36.025	1.497
15.341	38.158	1.497
14.846	37.47	1.497
15.297	52.31	1.497
13.538	68.914	1.497
14.971	86.128	1.497
10.331	32.367	1.497
12.769	59.108	1.663
8.419	79.381	1.377
13.551	55.477	1.377
15.499	72.225	1.377
9.598	76.377	1.377
16.105	51.513	1.526
16.239	35.612	1.268
13.238	25.306	1.444
10.584	77.28	1.497
12.612	57.033	1.497
12.608	81.314	1.497
15.914	41.212	1.373
12.838	50.489	1.373
14.112	58.828	1.435
12.515	83.682	1.465
13.092	30.227	1.465
14.386	73.586	1.438
11.301	73.348	1.438
14.142	75.409	1.649
16.269	16.525	1.472
15.372	12.208	1.472
15.476	37.879	1.431
14.089	56.234	1.431
16.695	47.025	1.403
13.777	87.512	1.401
15.14	59.343	1.571
9.759	24.686	1.571

10.394	39.895	1.43
13.362	66.273	1.481
13.816	56.971	1.481
12.275	68.447	1.508
14.702	69.727	1.432
15.555	58.109	1.527
11.347	53.24	1.527
10.551	43.478	1.318
15.346	83.059	1.611
12.969	64.501	1.335
12.664	58.671	1.067
13.919	76.765	1.351
15.05	42.481	1.5
15.053	10.438	1.334
14.461	12.911	1.334
9.891	12.977	1.334
12.859	28.864	1.334
14.092	77.565	1.334

9.958	16.588	1.334
11.949	71.113	1.524
15.476	63.749	1.5
14.102	44.002	1.566
14.098	44.239	1.566
9.656	63.634	1.566
11.794	35.986	1.498
11.557	76.576	1.6
14.28	10.069	1.6
14.639	80.123	1.598
14.529	83.501	1.409
14.511	82.479	1.409
15.808	67.223	1.409
13.752	66.358	1.434
12.904	60.08	1.562
13.829	43.305	1.562
12.513	40.795	1.562

10LWB 4.1

**zeta**	**sig zeta**	**rho-d (N/cm_)**	**Nd**
**361**	**10**	**1.26E+06**	**12697**

Ns	Ni	Dpar
19	19	1.519
29	14	1.487
21	13	1.563
136	59	1.534
151	56	1.474
47	25	1.491
43	25	1.533
15	7	1.495
77	38	1.651
35	12	1.485
44	23	1.4
54	25	1.456
28	19	1.413
48	22	1.412
51	28	1.642
50	19	1.329
134	57	1.433
117	59	1.525
63	30	0.571
24	11	1.407
100	38	1.468
26	18	1.548
129	58	1.732
126	50	1.564
170	69	1.572

**length**	**angle**	**Dpar**
13.823	44.139	1.5
12.629	77.776	1.5
16.279	44.255	1.388
11.866	77.847	1.5
12.784	65.505	1.5
14.515	73.797	1.5
14.151	61.504	1.5
14.155	87.246	1.5
12.885	55.49	1.5
13.932	58.526	1.657
11.42	69.232	1.35
13.839	51.392	1.5

12.45	78.951	1.5	12.97	40.664	1.497
11.694	69.981	1.481	14.041	70.324	1.497
12.746	46.258	1.556	12.399	57.897	1.5
15.014	35.166	1.525	13.594	20.792	1.5
15.86	55.849	1.5	12.968	20.72	1.5
12.446	26.762	1.297	13.997	78.731	1.515
13.62	36.009	1.423	14.325	73.223	1.526
13.9	30.036	1.629	12.749	59.415	1.75
14.794	47.146	1.5	12.326	54.742	1.5
10.09	64.385	1.5	12.372	56.376	1.343
13.505	56.832	1.5	11.845	53.254	1.761
13.376	59.545	1.406	12.888	66.323	1.594
15.009	66.083	1.5	12.424	66.114	1.426
15.186	86.482	1.641	13.608	61.658	1.426
13.267	54.809	1.421	16.016	59.697	1.553
13.553	83.104	1.321	13.322	35.644	1.553
13.927	57.438	1.5	15.678	1.948	1.639
13.427	40.93	1.312	13.951	45.528	1.639
12.586	68.855	1.5	13.71	16.901	1.293
13.345	48.503	1.413	11.48	82.262	1.293
12.639	88.2	1.5	13.769	77.557	1.391
15.302	71.432	1.5	13.793	7.464	1.499
11.716	83.733	1.5	13.933	25.272	1.169
8.469	47.394	1.5	14.411	68.521	1.442
14.386	48.672	1.5	14.29	52.019	1.442
13.769	80.458	1.5	13.462	61.074	1.442
13.045	74.739	1.5	13.108	66.256	1.422
12.939	72.595	1.5	12.41	25.669	1.422
13.556	80.792	1.5	15.486	29.001	1.422
13.527	80.27	1.5	14.674	49.533	1.417
13.823	80.484	1.572	12.539	43.271	1.719
11.724	83.937	1.5	13.498	51.614	1.719
12.599	69.159	1.5	13.961	86.182	1.596
13.733	49.621	1.597	11.812	43.702	1.596
14.214	56.783	1.5	12.413	16.179	1.596
14.069	56.108	1.5	15.089	1.278	1.428
13.174	41.416	1.5	13.266	17.264	1.428
15.521	64.665	1.5	14.32	68.785	1.575
12.899	48.121	1.5	13.314	26.287	1.575
12.614	45.043	1.551	14.335	88.637	1.575
12.212	80.84	1.939	14.979	63.154	1.575
12.614	67.722	1.939			

10LWB 4.3

zeta	sig zeta	rho-d (N/cm_)	Nd
**361**	**10**	**1.30E+06**	**12697**

Ns	Ni	Dpar
7	13	1.52
18	20	1.336
0	1	1.3
0	1	1.494
3	3	1.35
10	23	1.406
4	11	1.488
0	7	1.34
8	19	1.512
13	27	1.463
21	62	1.54
3	10	1.184
4	18	1.255
0	3	1.34
8	27	1.218
2	5	1.227
2	7	1.34
4	18	1.429
4	13	1.266
2	9	1.564
6	12	1.297
2	7	1.34
8	29	1.385
4	16	1.38
6	6	1.209
8	23	1.321
18	55	1.26
4	26	1.432
10	27	1.491
5	14	1.34
5	17	1.136
0	12	1.3
2	9	1.455
6	20	1.315
9	31	1.272
7	19	1.152

**length**	**angle**	**Dpar**
12.184	70.556	1.281
14.443	74.205	1.175
12.59	75.128	1.175
13.761	53.233	1.348
14.813	11.403	1.513
15.181	26.865	1.513
13.495	35.587	1.513
13.609	42.481	1.394
15.711	50.91	1.471
14.819	46.666	1.279
7.811	88.524	1.279
14.199	33.462	1.279
13.734	56.735	1.455
14.009	26.054	1.308
16.08	64.539	1.456
9.262	29.103	1.456
14.375	62.241	1.415
15.294	55.221	1.098
13.711	63.18	1.923
16.495	51.128	1.923
12.115	60.038	1.655
12.394	19.377	1.655
13.214	15.105	1.3
13.884	79.267	1.654
12.398	65.227	1.386
14.583	84.17	1.386
14.031	29.819	1.278
13.837	47.246	1.081
15.131	32.109	1.423
13.118	86.744	1.423
13.913	79.918	1.52
11.69	83.377	1.522
13.468	62.312	1.334
6.079	79.486	1.585
11.223	31.761	1.585
8.838	50.862	1.585
12.111	88.815	1.516

16.101	35.98	1.405
14.047	68.641	1.474
13.79	13.597	1.617
9.881	52.553	1.608
14.35	45.968	1.608
12.485	71.145	1.124
14.541	9.406	1.37
13.948	42.186	1.366
14.566	11.274	1.541
12.766	25.515	1.541
14.144	84.351	1.357
13.709	87.754	1.496
13.761	86.419	1.331
14.137	26.927	1.705
15.882	33.929	1.55
15.791	82.451	1.186
13.003	50.928	1.63
12.163	46.752	1.63
14.472	84.188	1.63
7.048	70.587	1.63
10.671	49.355	1.521
11.79	64.113	1.371
15.949	84.979	1.445

13.526	82.547	1.445
10.008	87.252	1.437
13.269	63.386	1.366
12.545	68.719	1.366
16.107	32.342	1.107
15.049	13.328	1.107
11.79	12.106	1.349
13.113	87.95	1.35
13.966	71.043	1.389
8.548	44.734	1.389
8.724	40.188	1.389
12.13	66.034	1.389
12.788	36.064	1.389
6.381	76.774	1.389
13.067	16.622	1.389
12.826	39.05	1.389
12.734	35.891	1.389
13.656	31.17	1.358
13.037	39.491	1.277
14.48	13.91	1.277
7.654	28.579	1.277
12.038	88.509	1.37

10lWB 4.5

**zeta**	**sig zeta**	**rho-d (N/cm≤)**	**Nd**
**361**	**10**	**1.33E+06**	**12697**
Ns	Ni	Dpar	
135	61	1.344	
32	22	1.366	
30	14	1.385	
62	33	1.4	
72	31	1.457	
170	130	1.469	
40	18	1.525	
29	17	1.545	
214	145	1.555	
64	34	1.564	
63	36	1.586	
61	29	1.587	
60	31	1.628	

84	50	1.65
53	31	1.71
14	16	1.748
35	25	1.44
41	15	1.8
42	17	1.64
27	22	1.5
59	31	1.42
18	10	1.42
41	30	1.37
25	16	1.53
40	18	1.45

**length**	**angle**	**Dpar**
12.811	56.354	1.63
12.488	53.996	1.63

11.52	75.138	1.63	12.614	83.76	1.639
14.373	8.675	1.63	11.823	61.518	1.881
12.714	12.992	1.63	10.995	85.395	1.881
12.389	72.318	1.63	10.366	77.483	1.881
14.474	65.934	1.63	11.263	61.86	1.881
14.425	43.014	1.68	14.886	72.303	1.881
13.84	67.255	1.68	10.657	83.873	1.881
9.721	83.195	1.68	12.188	58.994	1.881
15.077	19.693	1.68	14.746	57.03	1.881
12.673	51.37	1.68	12.498	53.158	1.881
13.69	60.31	1.633	10.803	44.69	1.881
11.813	63.718	1.401	14.569	82.667	1.685
12.822	64.486	1.533	13.228	21.947	1.685
12.902	54.568	1.533	10.799	62.438	1.827
11.063	78.571	1.533	16.261	40.066	1.827
15.887	39.876	1.476	12.116	51.728	1.827
12.685	78.337	1.476	10.317	75.344	1.827
14.128	76.226	1.51	14.555	48.246	1.827
15.258	44.942	1.51	13.589	83.421	1.495
10.169	70.683	1.51	7.439	51.483	1.495
10.437	80.117	1.521	13.323	62.076	1.495
15.175	46.994	1.744	10.524	79.517	1.495
13.056	76.604	1.744	10.736	74.431	1.495
14.416	74.247	1.744	11.027	74.529	1.743
11.886	72.139	1.744	11.868	55.319	1.743
11.515	65.963	1.479	12.253	80.572	1.743
14.545	34.949	1.479	13.837	73.008	1.743
11.744	69.108	1.594	11.363	74.062	1.743
13.851	33.13	1.594	10.136	56.41	1.743
13.102	31.503	1.594	12.939	47.61	1.565
13.3	69.615	1.512	12.814	61.501	1.565
14.014	17.378	1.512	12.08	70.022	1.565
13.189	13.593	1.31	13.472	26.794	1.565
13.94	10.723	1.31	10.999	75.18	1.565
15.601	63.124	1.501	13.296	25.94	1.565
13.9	22.203	1.501	5.289	79.643	1.579
11.589	69.908	1.501	14.076	35.528	1.579
13.481	46.983	1.501	11.589	46.078	1.579
14.487	64.256	1.413	12.067	56.506	1.347
14.166	64.373	1.639	12.223	82.202	1.686
11.767	76.689	1.639	12.67	50.877	1.686
12.092	55.351	1.639	12.326	72.803	1.686
13.406	66.315	1.639	12.983	84.286	1.686

10.221	64.336	1.686
12.994	57.743	1.686
12.378	46.097	1.686
13.914	25.475	1.686
12.823	53.514	1.686
14.116	21.455	1.686
12.597	80.188	1.686

10.972	61.047	1.686
12.725	41.994	1.686
12.643	80.63	1.686
12.163	78.112	1.506
12.236	79.425	1.717

10LWA 8.1

**zeta**	**sig zeta**	**rho-d (N/cm≤)**	
**361**	**10**	**1.41E+06**	**12697**

Ns	Ni	Dpar
124	93	1.804
84	68	1.542
56	35	1.831
80	65	1.441
90	52	1.603
217	139	1.893
130	102	1.761
123	78	1.811
130	83	1.836
131	74	1.688
180	78	1.848
143	110	1.74
204	124	1.73
47	28	1.73
244	141	1.73
78	47	1.73
112	88	1.73
202	115	1.73
141	82	1.73
85	66	1.73
227	183	1.73
295	169	1.73
167	104	1.73
76	55	1.73
84	72	1.73

**length**	**angle**	**Dpar**
13.753	59.452	1.793
11.055	35.665	1.793
13.239	46.718	1.793
6.965	62.587	1.273
13.542	85.603	1.273
10.836	35.657	1.273
15.138	73.811	1.726
11.913	59.847	1.726
10.427	51.433	1.726
10.989	28.457	1.726
11.964	16.742	1.61
12.425	47.647	1.61
10.384	86.389	1.534
12.768	22.945	1.534
13.244	51.222	1.64
14.226	37.149	1.64
10.214	70.115	1.64
8.248	56.799	1.64
10.379	81.433	1.276
13.93	77.46	1.544
10.341	56.742	1.544
8.651	67.502	1.544
9.022	84.722	1.667
16.915	72.234	1.667
14.535	75.185	1.667
13.876	53.732	1.667
14.78	60.809	1.667
14.91	67.296	1.407
12.547	43.682	1.776
10.692	65.825	1.776
11.966	40.7	1.776
13.618	46.317	1.583
15.171	77.94	1.583
13.496	49.74	1.583
12.498	38.47	1.663

12.609	62.818	1.663
13.621	58.915	1.663
9.288	75.858	1.663
11.042	68.87	1.663
13.334	58.542	1.663
11.287	77.783	1.634
9.649	10.887	1.634
11.231	71.221	1.634
10.068	71.949	1.712
13.6	68.937	1.712
12.485	12.649	1.712
10.658	63.756	1.712
14.858	81.793	1.712
14.926	3.803	1.712
12.978	71.742	1.712
7.929	40.638	1.712
9.821	77.38	1.712
10.671	68.842	1.693
14.231	45.805	1.693
10.435	83.541	1.286
12.191	7.425	1.286
15.241	81.837	1.286
14.73	12.993	1.286
7.731	72.664	1.286
10.997	72.192	1.286
11.852	56.55	1.286
9.814	53.515	1.286
14.021	85.263	1.286
9.662	60.72	1.286
10.035	88.463	1.286
9.926	85.803	1.505
10.815	54.528	1.69
12.346	84.874	1.69
13.618	77.562	1.69
11.478	29.687	1.69
13.623	53.846	1.69
11.582	72.124	1.596
11.654	67.01	1.596
12.689	73.959	1.596
12.645	27.188	1.596
12.332	25.16	1.596
15.482	77.078	1.627
11.582	80.122	1.627

10.302	72.143	1.627
14.782	47.781	1.627
13.5	45.967	1.627
11.341	56.956	1.627
9.453	75.446	1.6
10.254	70.306	1.6
13.774	6.702	1.862
13.413	45.673	1.633
14.134	41.736	1.633
11.323	80.686	1.577
12.462	38.69	1.577
11.228	59.879	1.577
9.319	76.388	1.577
11.094	79.106	1.764
13.726	87.244	1.764
10.019	88.223	1.764
14.879	19.963	1.764
10.648	72.523	1.775
12.13	41.526	1.775
10.303	36.259	1.775
11.005	70.199	1.775
10.039	67.099	1.728
15.631	67.838	1.728
14.782	51.726	1.848
9.562	67.17	1.617
11.155	49.144	1.617
11.414	30.803	1.617
13.622	68.598	1.667
10.937	71.81	1.667
15.169	46.671	1.768
12.848	75.427	1.768
11.699	61.839	1.768
10.216	57.485	1.768
13.825	72.76	1.768
9.264	81.291	1.768
14.987	65.088	1.768
13.526	46.487	1.768
14.63	43.738	1.768
12.012	86.903	1.768
13.025	86.981	1.522
11.711	54.492	1.522
12.188	52.638	1.522
11.066	51.028	1.892

14.367	29.696	1.892
12.985	75.367	1.578
12.217	47.163	1.578
10.525	71.44	1.578
13.235	76.878	1.652
14.88	45.311	1.652
13.842	85.304	1.652
13.487	48.999	1.697
12.238	68.283	1.697
9.03	66.778	1.697
11.834	48.196	1.697
10.788	67.313	1.697
12.587	51.823	1.697
10.769	56.713	1.726
15.202	49.638	1.726
14.454	44.45	1.726
12.429	34.537	1.726
12.457	43.95	1.726
12.853	43.61	1.726
9.468	69.11	1.726
14.294	58.194	1.726
11.117	50.907	1.726
10.601	64.772	1.726

9.879	70.263	1.543
11.887	81.256	1.543
12.489	61.963	1.654
11.654	14.718	1.654
11.25	75.219	1.654
15.099	88.942	1.654
14.819	62.719	1.654
14.512	52.485	1.654
14.423	76.9	1.831
12.975	65.474	1.831
12.255	88.845	1.831
13.306	78.647	1.831
5.864	79.173	1.831
11.564	88.348	1.831
11.641	71.933	1.831
12.108	29.601	1.831
11.839	42.385	1.831
13.571	65.167	1.831
11.507	37.318	1.584
11.527	38.417	1.584
10.71	83.541	1.584

10LWA 11.1

**zeta**	**sig zeta**	**rho-d (N/cm≤)**	**Nd**
**361**	**10**	**1.43E+06**	**12697**

Ns	Ni	Dpar
111	222	1.368
24	60	1.406
92	213	1.555
100	170	1.567
68	116	1.604
91	148	1.633
29	69	1.637
17	25	1.641
44	91	1.649
120	219	1.696
50	123	1.701
38	74	1.704
47	99	1.704
72	185	1.707
74	121	1.728
108	238	1.733
129	221	1.744
222	372	1.754
69	120	1.762
96	162	1.765
86	166	1.797
69	157	1.822
64	109	1.885
79	196	1.946
35	80	1.976

**length**	**angle**	**Dpar**
9.817	84.298	1.635

10.763	13.059	1.635	14.703	67.261	1.649
16.877	36.226	1.635	8.653	33.765	1.649
16.55	37.257	1.635	12.476	64.853	1.643
11.509	83.851	1.635	9.887	69.964	1.643
12.897	64.232	1.71	12.752	81.393	1.643
12.52	59.383	1.827	14.226	50.273	1.823
11.302	74.952	1.669	9.647	39.472	1.68
13.178	70.661	1.83	14.743	85.407	1.757
14.414	28.047	1.83	14.526	37.989	1.796
15.22	27.593	1.734	16.541	44.875	1.796
12.947	15.989	1.734	16.569	69.065	1.796
16.035	48.998	1.584	14.054	54.295	1.644
13.844	56.536	1.584	13.103	9.312	1.644
15.225	41.458	1.593	14.872	33.612	1.384
15.581	36.478	1.593	15.934	14.149	1.384
13.782	8.258	1.377	10.921	73.935	1.384
13.043	73.502	1.568	13.658	18.234	1.289
12.856	54.07	1.839	11.244	75.759	1.444
14.995	14.405	1.839	10.461	50.075	1.444
12.565	23.589	1.649	15.46	49.363	1.444
13.904	89.995	1.649	12.468	84.547	1.444
11.877	65.738	1.649	13.361	78.118	1.444
14.929	5.267	1.559	11.806	68.53	1.444
14.087	54.723	1.679	15.067	63.901	1.444
11.698	35.319	1.679	11.75	62.951	1.444
13.868	34.278	1.679	15.029	77.874	1.444
13.136	62.86	1.831	11.331	75.103	1.756
14.614	80.939	1.628	13.543	77.929	1.756
14.123	53.254	1.628	12.252	86.267	1.913
9.823	69.75	1.628	14.245	32.267	1.913
11.867	61.137	1.733	13.323	55.92	1.913
14.907	15.019	1.733	12.943	28.803	1.913
10.606	22.968	1.733	14.63	70.166	1.616
11.362	9.159	1.733	12.628	59.801	1.827
15.234	16.995	1.691	13.826	89.464	1.827
12.979	39.988	1.691	13.182	1.124	1.791
14.871	2.052	1.691	13.142	27.414	1.791
14.059	52.99	1.691	10.132	75.469	1.805
13.625	45.343	1.691	11.871	46.656	1.805
11.667	78.045	1.662	15.12	77.027	1.805
12.985	23.421	1.885	14.049	16.059	1.65
11.015	62.526	1.729	12.492	73.626	1.65
8.613	21.328	1.71	14.988	38.368	1.65

13.768	20.628	1.65	12.671	85.498	1.984
7.082	77.002	2.065	16.55	69.57	1.54
17.409	63.402	1.816	14.93	45.274	1.54
8.109	39.559	1.816	16.703	45.011	1.54
15.02	45.003	1.816	14.463	34.969	1.54
13.028	48.979	1.816	15.184	57.858	1.7
13.617	48.715	1.618	10.563	17.657	1.7
13.077	55.785	1.618	16.852	39.924	1.7
11.797	42.525	1.618	14.906	13.756	1.7
15.011	50.584	1.618	16.1	42.36	1.7
14.102	50.564	1.987	11.274	86.658	1.7
13.514	54.893	1.987	10.793	69.404	2.002
15.072	14.12	1.984	13.51	41.195	2.002
13.424	33.84	1.984			

Supplementary Data Table S3: Apatite (U-Th)/He Data

Table S3a: Apatite (U-Th)/He data for East Antarctic cobbles, central Transantarctic Mountains region

Sample	Dim Mass (µg)	rs (µm)	l (µm)	4He (nmol/g)	U (ppm)	Th (ppm)	Sm (ppm)	eU	Raw Age (Ma)	FT	Corr. Age (Ma)	Full Unc. (Ma)	Analytic Unc. (Ma)
Mount Sirius (84°07.976'S, 163°15.121'E, 2160 m)													
**10MSA3.5**													
10MSA3.5_a01	2.54	52.60	171.3	6.920	11.58	9.51	62.04	13.8	88.92	0.732	120.0	8.6	1.2
10MSA3.5_a2	1.90	47.25	156.8	9.797	21.04	19.52	78.31	25.6	68.74	0.702	97.0	6.9	0.9
10MSA3.5_a4	1.15	41.07	129.5	5.008	13.32	9.15	55.21	15.5	58.04	0.663	86.4	6.8	1.4
								Mean age ± 1 σ (% std. dev.) of all: 101 ± 17 Ma (2%)					
Lone Wolf Nunataks (Site A [LWA]: 81°20.186'S, 152°42.461'E, 1590 m; Site B [LWB]: 81°19.545'S, 153°01.445'E, 1595 m)													
**10LWB4.1**													
10LWB4.1_a1	2.99	55.85	190.9	3.812	4.85	0.86	58.20	5.1	126.50	0.746	*165.6*	13.0	3.2
10LWB4.1_a2	1.23	39.14	149.6	45.724	24.29	41.00	124.56	33.9	238.05	*0.636*	366.3	34.9	3.9
10LWB4.1_a3	1.36	40.83	154.2	21.032	12.31	4.12	89.41	13.3	272.06	0.664	398.2	34.0	5.8
								Mean age ± 1 σ (% std. dev.) of all: 310 ± 126 Ma (40%)					
								Mean age ± 1 σ (% std. dev.) of grains 2 and 3: 382 ± 23 Ma (6%)					
**10LWB4.3**													
10LWB4.3_a1	8.92	80.94	251.3	0.235	0.26	1.43	1.92	0.6	70.64	0.812	86.5	7.5	3.6
10LWB4.3_a2	1.08	39.18	128.3	0.538	1.24	8.11	3.15	3.1	31.31	*0.619*	50.3	5.8	2.4
10LWB4.3_a3	0.97	39.02	112.9	0.576	0.51	3.67	1.04	1.4	76.79	*0.620*	*123.2*	24.3	14.7
								Mean age ± 1 σ (% std. dev.) of all: 87 ± 36 Ma (42%)					
								Mean age ± 1 σ (% std. dev.) of grains 1 and 2: 68 ± 26 Ma (37%)					
**10LWB4.5**													
10LWB4.5_a2	2.26	47.59	151.9	5.028	16.09	4.19	26.59	17.1	53.69	0.713	74.9	5.2	0.9
10LWB4.5_a3	1.06	39.57	124.8	56.710	40.90	4.11	35.47	41.9	244.14	0.657	366.5	30.0	3.0
10LWB4.5_a3	1.58	40.76	148.5	27.816	32.94	4.55	38.01	34.0	148.41	0.665	221.0		9.0
								Mean age ± 1 σ (% std. dev.) of all: 220 ± 146 Ma (66%)					
**10LWA8.1**													
10LWA8.1_a1	7.15	65.59	267.8	127.515	63.98	3.62	8.89	64.8	352.83	0.789	*443.2*	31.1	4.4
10LWA8.1_a2	4.14	62.61	218.0	49.256	46.21	3.27	13.59	47.0	190.79	0.777	*244.2*	17.4	2.3
10LWA8.1_a3	1.91	45.12	136.1	24.562	39.98	5.71	6.71	41.3	109.10	0.698	155.6		6.2
10LWA8.1_a4	2.38	48.38	103.0	22.387	37.27	3.44	8.13	38.1	107.88	0.721	148.9		9.8
								Mean age ± 1 σ (% std. dev.) of all: 248 ± 137 Ma (55%)					
								Mean age ± 1 σ (% std. dev.) of grains 3 and 4: 152 ± 5 Ma (3%)					
**10LWA11-1**													
10LWA11-1_a1	1.32	41.40	140.6	21.333	42.61	125.15	26.07	72.0	54.41	*0.648*	83.6	7.0	0.5
10LWA11-1_a2	1.13	38.41	102.6	15.791	35.71	62.01	12.94	50.3	57.76	*0.635*	90.5	8.0	0.5
10LWA11-1_a3	1.21	39.95	139.8	14.530	39.99	76.09	12.83	57.9	46.23	*0.641*	71.8	6.6	0.6
10LWA11-1_a4	1.45	38.31	158.8	18.581	31.37	129.12	12.69	61.7	55.34	*0.616*	89.6	12.0	0.6
10LWA11-1_a5	1.78	43.58	136.5	14.486	29.16	51.79	10.22	41.3	64.44	0.674	95.3	7.0	0.7
								Mean age ± 1 σ (% std. dev.) of all: 86 ± 9 Ma (10%)					

Analyses were completed by CU TRaIL. Dim. Mass = dimensional mass of grain calculated from crystal volume and average apatite density; r = radius of a sphere with equivalent surface area to volume ratio as the grain; l = longest dimension of the grain. Concentrations of U, Th and Sm measured via isotope dilution on an ICP-MS. eU is the effective Uranium, calculated as [U] + 0.235[Th] e.g., [12]. Grains were degassed by heating with a laser to determine the amount of ^{4}He (nmol/g) in the grain. Alpha ejection correction (F_T) is a measure of the amount of He ejected from the crystal, values <0.65 (italicized, grey) indicate that a significant amount of He was ejected [13]. Ages and F_T were calculated using methods described in Ketcham [14]. Raw Age = age calculated from isotope concentrations, without F_T correction; Corr. Age = age calculated from isotope concentrations, with F_T correction;

Corrected ages in red italics are considered outliers as these ages plot outside the envelope of acceptable T-t paths on the inverse thermal models. Full uncertainty is ± 1σ and incorporates F_T uncertainty. Summarized in bold italics, is the mean age of the single-grain corr. ages with 1σ error (1 standard deviation) and in parenthesis is the coefficient of variation (% std. dev.). In parenthesis is the coefficient of variation (% std. dev.), calculated from the ratio of the standard deviation to the mean. Coefficient of variation is used to show the variation of single-grain ages from the mean age. Mean ages are calculated with outlier single grain ages (in red) and without identified outlier single grain ages (in black). This follows the method of Ault et al. [15].

Supplementary Data Table S3b: CU TRaIL notes on apatite grains used for apatite (U-Th)/He analyses on East Antarctic cobbles

Grain #	Correc. Age (Ma)	Full Unc. (Ma)	Notes
Mount Sirius (84°07.976'S, 163°15.121'E, 2160 m)			
**10MSA3.5**			
10MSA3.5_a1	120.0	8.6	Big, some surface pits but clear, euh, perhaps one end is a little ground?
10MSA3.5_a2	97.0	6.9	med, clear, euhedral with faces but a little rounded, no incs
10MSA3.5_a4	86.4	6.8	clear, missing one term but euh with xtal faces
	Mean age ± 1 σ (% std. dev.) of all: 101 ± 17 Ma (2%)		
Lone Wolf Nunataks (Site A [LWA]: 81°20.186'S, 152°42.461'E, 1590 m; Site B [LWB]: 81°19.545'S, 153°01.445'E, 1595 m)			
**10LWB4.1**			
10LWB4.1_a1	*165.6*	13.0	medium sized, rounded, clear inside, but oval shape
10LWB4.1_a2	366.3	34.9	small, ground but xtal faces are clearly visible
10LWB4.1_a3	398.2	34.0	small, ground but xtal faces are clearly visible
	Mean age ± 1 σ (% std. dev.) of all: 310 ± 126 Ma (40%)		
	Mean age ± 1 σ (% std. dev.) of grains 2 and 3: 382 ± 23 Ma (6%)		
**10LWB4.3**			
10LWB4.3_a1	86.5	7.5	Huge grain, clear with some surface rounding chips. No visible incs though
10LWB4.3_a2	50.3	5.8	small, beautiful xtal, no incs, good form, one corner chipped
10LWB4.3_a3	*123.2*	24.3	med to small, ground outside, looks clear though
	Mean age ± 1 σ (% std. dev.) of all: 87 ± 36 Ma (42%)		
	Mean age ± 1 σ (% std. dev.) of grains 1 and 2: 68 ± 26 Ma (37%)		
**10LWB4.5**			
10LWB4.5_a2			med, 1 term, clear, no incs, xtal faces clear
10LWB4.5_a3			small, clear, very pristine looking with a small pit on a face
10LWB4.5_a3			no visible inclusions, one side has surface marks that may obscure a small inclusion, very rounded, smallis
	Mean age ± 1 σ (% std. dev.) of all: 220 ± 146 Ma (66%)		
**10LWA8.1**			
10LWA8.1_a1			big and clear, no incs, one end chipped, one term perfect
10LWA8.1_a2			big, rounded but I can see xtal faces, two terms, a few surface marks but no incs
10LWA8.1_a3			no visible inclusions, some faint fractures, one broken tip, sides clearly visible, very minor surface marks
10LWA8.1_a4			no obvious inclusions, broken tip, one very faint mark that looks surficial, also faint fractures
	Mean age ± 1 σ (% std. dev.) of all: 248 ± 137 Ma (55%)		
	Mean age ± 1 σ (% std. dev.) of grains 3 and 4: 152 ± 5 Ma (3%)		
**10LWA11.1**			
10LWA11-1_a01	83.6	7.0	broken end, no visible inclusions, clear, smooth faces, edges somewhat pocked
10LWA11-1_a02	90.5	8.0	broken end, no visible inclusions, clear, smooth faces, edges somewhat pocked
10LWA11-1_a03	71.8	6.6	looks like 2 pry, some minor surface fractures but mosly clear and smooth. No visible inclusions
10LWA11-1_a04	89.6	12.0	broken end, no visible inclusions, clear, smooth faces, edges somewhat pocked
10LWA11-1_a05	95.3	7.0	broken end I think, no visible inclusions, clear, smooth faces, some fractured corners
	Mean age ± 1 σ (% std. dev.) of all: 86 ± 9 Ma (10%)		

Supplementary Data S4: Apatite (U-Th)/He data trend analysis

Single-grain age plots (age vs eU, age vs grain size)
Group 1 samples: Solid marker symbols lie within the good and acceptable T-t model paths (see below) and are used to determine mean AHe ages, whereas hollow marker symbols lie outside the good and acceptable T-t model paths and are not included in mean age determinations. Samples 10LWB 4.5 and 10LWA 8.1 He single-grain ages (SGA) vs. effective urainium [eU] display a positive trend (higher [eU] grains = older ages as would be expected). For AHe ages vs grain size (approximated by "r": radius of a sphere with an equivalent surface area to volume ratio as the apatite grain) sample 10LWA 8.1 has a positive trend (larger grains = older ages, as expected. Such trends which are to be expected due to prolonged residence within a AHe partial retention zone are taken into account during inverse modeling by selection of the RDAAM calibration in the models [12].

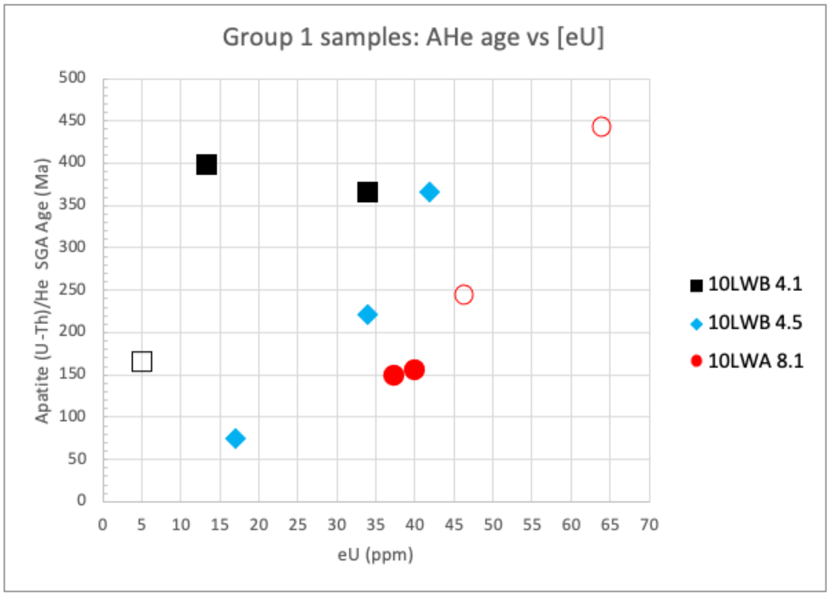

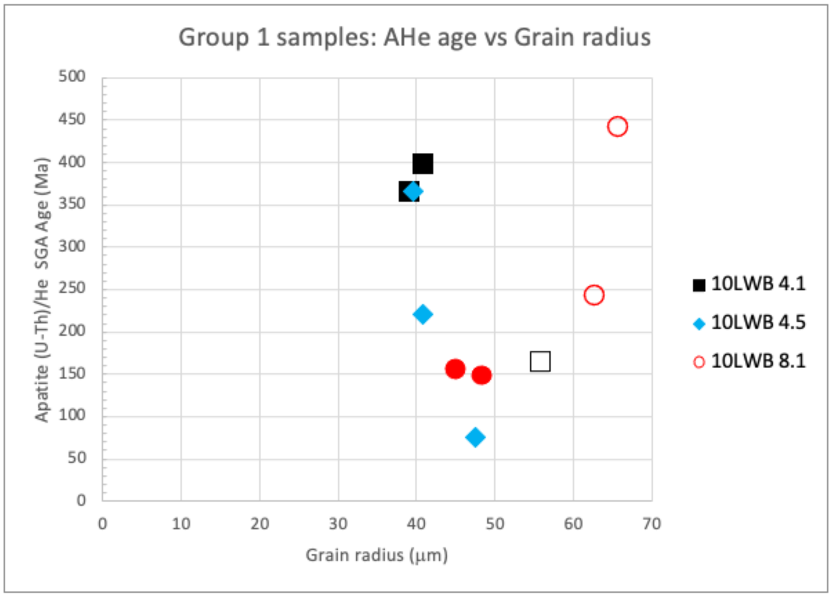

Group 2 samples: Solid marker symbols lie within the good and acceptable T-t model paths (see below) and are used to determine mean AHe ages, whereas hollow marker symbols lie outside the good and acceptable T-t model paths and are not included in mean age determinations. There are no positive trends for SGA's vs. [eU] or grain size from each sample. As compared to group 1 samples, group 2 samples spent considerably less time within an apatite partial retention zone, thus differences in grain size (= the effective diffusion domain) or [eU] have not resulted in a noticeable spread of SGA's.

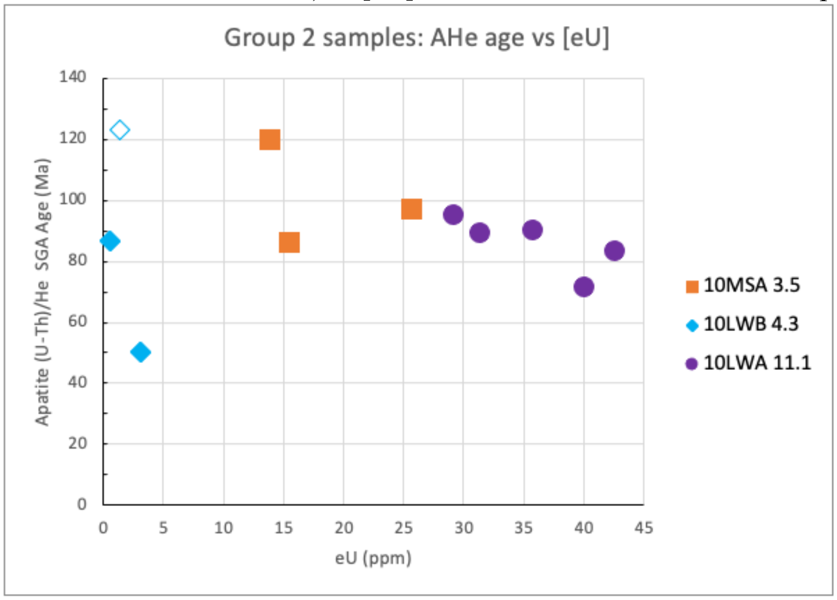

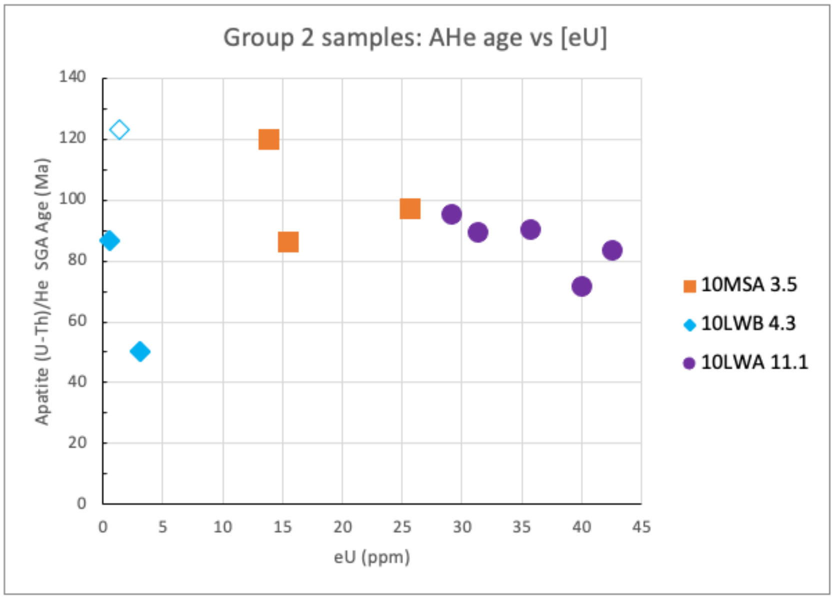
Group 2 samples: AHe age vs [eU]
Apatite (U-Th)/He SGA Age (Ma)
140
120
100
80
60
40
20
0
0
5
10
15
20
25
30
35
40
45
eU (ppm)
10MSA 3.5
10LWB 4.3
10LWA 11.1

Supplementary Data S5: INVERSE THERMAL MODELING

Inverse thermal modeling is a valuable tool to convert thermochronologic data into interpretable information and it is crucial to include information on modeling inputs and parameters so others may evaluate and reproduce the results e.g., Flowers et al. [16] recommends a model input table to standardized thermochronological modeling reporting. The tables above include information on the apatite fission track (AFT) and apatite U-Th/He (AHe) data, geologic constraints, and system- and model-specific parameters.

The program HeFTy (v1.9.3) was used to generate inverse thermal models[17, 18] that are depicted in Figure 3. Those models include: time-temperature (T-t) path envelopes (good paths in magenta and acceptable paths in green), best-fit path (black line), weighted mean path (thick blue line). Constraint boxes for T-t paths are started “loose” – and designed to allow the Monte Carlo models to explore all available T-t space, allowing both cooling and heating paths.

Two of the samples with AHe data contained single-grain age variation with significantly older outliers and one sample had one young grain that we identified as an outlier (Figure 3 in the paper, Supplementary Table S3a). Adding AHe data to models generally yielded slightly better constrained “good” and “acceptable” T-t path envelopes through the AHe partial retention zone (~30-90 °C). Given the significant variation of AHe single-grain ages and lack of kinetic parameter for that method as compared to apatite fission track thermochronology, when the model was unable to produce paths with both AFT and AHe data, AFT data was used exclusively. AFT ages are placed on the weighted mean path (thick blue line) whereas the individual AHe ages are placed on appropriate closure temperatures for AHe ages from each sample, which were determined based on grain size and cooling rate e.g., [19]. In general, larger grains will have higher closure temperatures and samples that cool quickly will also have higher closure temperatures. The approximate cooling rate for the spread of AHe ages was determined using inverse thermal model output (e.g., – for sample 10LWB4.5 the cooling rate for the spread of the AHe ages (75 – 367 Ma) is ~0.1°C/Myr, the radius of each grain is ~40 µm and hence the closure temperature is ~40°C. The long residence in the partial annealing zone is likely to have accentuated the single grain age spread, and in this case all three grains despite the age spread lie within the good and acceptable model T-t envelopes. For sample 10LWB4.3 the cooling rate for the spread of the AHe ages (50 – 123 Ma) is ~1°C/Myr, the radius of grain is from ~40-80 µm and hence the closure temperature is ~55°C. The oldest sample is considered an outlier, whereas the other two samples lie within the good and acceptable model T-t envelopes

Thermal history model input table for East Antarctic cobbles

1. Thermochronologic data

Samples and data used in models

	Model							Data source	This table is summative only	
	MSA-2.3	MSA-3.5	LWB-4.1	LWB-4.3	LWB-4.5	LWA-8.1	LWA-11.1			
AFT data										
MSA-2.3	x							Table S1		summative only, see raw data in Table S2b
MSA-3.5		x						Table S1		summative only, see raw data in Table S2b
LWB-4.1			x					Table S1		summative only, see raw data in Table S2b
LWB-4.3				x				Table S1		summative only, see raw data in Table S2b
LWB-4.5					x			Table S1		summative only, see raw data in Table S2b
LWA-8.1						x		Table S1		summative only, see raw data in Table S2b
LWA-11.1							x	Table S1		summative only, see raw data in Table S2b
AHe data										
MSA-2.3;	*							Table S3	yes	* no AHe data (not sufficent apatite)
MSA-3.5: a2, a4		x						Table S3	yes	
LWB-4.1: a3			x							
LWB-4.3: a2				x				Table S3	yes	
LWB-4.5: a3					x			Table S3	yes	
LWA-8.1: a3						x		Table S3	yes	
LWA-11.1: a5							x	Table S3	yes	

Data treatment, uncertainties, and other relevant constraints

AFT Data	*Zeta calibration: 361 ± 10 (PGF)*
	Additional information on data included in Supplementary Table S1 Analyses
AHe data	*completed at CU Trail by James Metcalf*
	Additional information on data and grain quality
	included in supplementary table S3 and S4

Other geochronology

	U-Pb zircon	Reference
10MSA-2.3	1410 ± 10 Ma	*Goodge et al.* [1]
10MSA-3.5	1508 ± 12 Ma	*Goodge et al.* [1]
10LWB-4.1	1865 ± 9 Ma	*Goodge et al.* [1]
10LWB-4.3	1448 ± 5 Ma	*Goodge et al.* [1]
10LWB-4.5	1848 ± 13 Ma	*Goodge et al.* [1]
10LWA-8.1	2015 ± 12 Ma	*Goodge et al.* [1]
10LWA-11.1	1213 ± 14 Ma	*Goodge et al.* [1]

2. Additional geologic information

Mean surface temperature –15 ± 10°C

All models started with initial constraint box 140-160°C, older than than the AFT age

3. System- and model-specific parameters

Model	*Modeling program: HeFTy v. 1.9.3*<br>*Statistical fitting criteria: Default. GOF values >0.05 are acceptable fits.*<br>GOF values >0.5 are good fit<br>*Ending condition: 5 or 10 good paths*
AFT	*Annealing model: Ketcham et al.* [18]<br>*C-axis projection: Ketcham et al.* [17]*, 5.0M; model used c-axis projected lengths Default initial mean track length: From Dpar (16.3 µm); length reduction in standard 0.893 Kinetic parameter: Dpar (µm), one kinetic population (per sample)*<br>*Dpar calibration for inverse HeFTy modeling: 0.997*<br>*Length calibration for inverse HeFTy modeling: 1.01*
AHe	*Calibration:* RDAAM, apatite *(Flowers et al.* [12] *; Precision,* Good<br>*Stopping distances:* Ketcham et al. [14]*, Alpha calculation:* Redistribution<br>*Age to report:* Corrected*;* Alpha age correction*: Ketcham et al.* [14]

References for Supplementary Data

1. Goodge JW, Fanning CM, Fisher CM, Vervoort JD. Proterozoic crustal evolution of central East Antarctica: Age and isotopic evidence from glacial igneous clasts, and links with Australia and Laurentia. *Precambrian Research* **299**, 151-176 (2017).
2. Hurford AJ, Green PF. The zeta age calibration of fission track dating. *Isotope Geoscience* **1**, 285-317 (1983).
3. Green PF. Comparison of zeta calibration baselines for fission-track dating of apatite, zircon and sphene. *Chemical Geology (Isotope Geoscience Section)* **58**, 1-22 (1985).
4. Green PF. A new look at statistics in fission track dating. *Nuclear Tracks and Radiation Measurements* **5**, 77-86 (1981).
5. Galbraith RF. On statistical models for fission track counts. *Journal of the International Association of Mathematical Geologists* **13**, 471-488 (1981).
6. Laslett GM, Gleadow AJW, Duddy IR. The relationship between fission track length and density in apatite. *Nuclear Tracks and Radiation Measurements* **9**, 29-38 (1984).
7. Galbraith R. Graphical display of estimates having differing standard errors. *Technometrics* **30**, 271-281 (1988).
8. Galbraith RF. The radial plot: graphical assessment of spread in ages. *International Journal of Radiation Applications and Instrumentation Part D Nuclear Tracks and Radiation Measurements* **17**, 207-214 (1990).
9. Vermeesch P. RadialPlotter: A Java application for fission track, luminescence and other radial plots. *Radiation Measurements* **44**, 409-410 (2009).
10. Vermeesch P. IsoplotR: A free and open toolbox for geochronology. *Geoscience Frontiers* **9**, 1479-1493 (2018).
11. Galbraith RF, Green PF. Estimating the component ages in a finite mixture. *Nuclear Tracks and Radiation Measurements* **17**, 197-206 (1990).
12. Flowers RM, Ketcham RA, Shuster DL, Farley KA. Apatite (U-Th)/He thermochronometry using a radiation damage accumulation and annealing model. *Geochimica et Cosmochimica Acta* **73**, 2347-2365 (2009).
13. Farley KA, Wolf RA, Silver LT. The effects of long alpha-stopping distances on (U-Th)/He ages. *Geochimica et Cosmochimica Acta* **60**, 4223-4229 (1996).
14. Ketcham RA, Gautheron C, Tassan-Got L. Accounting for long alpha-particle stopping distances in (U–Th–Sm)/He geochronology: Refinement of the baseline case. *Geochimica et Cosmochimica Acta* **75**, 7779-7791 (2011).
15. Ault AK, Flowers RM, Bowring SA. Phanerozoic surface history of the Slave craton. *Tectonics* **32**, 1066-1083 (2013).
16. Flowers RM, Farley KA, Ketcham RA. A reporting protocol for thermochronologic modeling illustrated with data from the Grand Canyon. *Earth and Planetary Science Letters* **432**, 425-435 (2015).

17. Ketcham R, Carter A, Donelick R, Barbarand J, Hurford A. Improved measurement of fission-track annealing in apatite using c-axis projection. *American Mineralogist* **92**, 789 (2007).

18. Ketcham RA. Forward and inverse modeling of low temperature thermochronometry data. In: *Low-Temperature thermochronology: Techniques, Interpretations and Applications* (eds Reiners PW, Ehlers TA). Mineralogical Society of America, Geochemical Society (2005).

19. Reiners PW, Brandon MT. Using thermochronology to understand orogenic erosion. *Annual Review of Earth and Planetary Sciences* **34**, 419-466 (2006).
